# Real‐Time Feedback Strategically Regulates Optoelectronics for Customized Optogenetic Spinal Cord Regeneration

**DOI:** 10.1002/EXP.20250138

**Published:** 2026-04-09

**Authors:** Kaishun Xia, Runze Hu, Xiaopeng Zhou, Jiangjie Chen, Xinmao You, Jingkai Wang, Hao Li, Yiqing Tao, Chao Yu, Haibin Xu, Yuang Zhang, Kesi Shi, Yi Li, Chenggui Wang, Fangcai Li, Chengzhen Liang, Ying Chen, Qixin Chen

**Affiliations:** ^1^ Department of Orthopedics The Second Affiliated Hospital School of Medicine Zhejiang University Hangzhou Zhejiang P. R. China; ^2^ Orthopedics Research Institute of Zhejiang University Zhejiang University Hangzhou Zhejiang P. R. China; ^3^ Key Laboratory of Motor System Disease Research and Precision Therapy of Zhejiang Province Hangzhou Zhejiang P. R. China; ^4^ Clinical Research Center of Motor System Disease of Zhejiang Province Hangzhou Zhejiang P. R. China; ^5^ Institute of Flexible Electronics Technology of THU Zhejiang Jiaxing Zhejiang P. R. China; ^6^ Department of Internal Medicine 3 Rheumatology and Immunology Friedrich‐Alexander University (FAU) Erlangen‐Nürnberg and Universitätsklinikum Erlangen Erlangen Germany; ^7^ Deutsches Zentrum Immuntherapie Friedrich‐Alexander University (FAU) Erlangen‐Nürnberg and Universitätsklinikum Erlangen Erlangen Germany; ^8^ Department of Orthopedics Yuyao People's Hospital Yuyao Zhejiang P. R. China; ^9^ Department of Orthopedics The Second Affiliated Hospital and Yuying Children's Hospital, Wenzhou Medical University Wenzhou Zhejiang P. R. China

**Keywords:** customized regulation strategy, multifunction, optogenetic, real‐time feedback, spinal cord injury

## Abstract

Previous optogenetic bioelectronic systems have enabled a highly selective way of modulating neural populations by delivering a certain wavelength of light to engage with exogenously expressed light‐sensitive proteins, which lay the foundation of therapeutic interventions of neural circuits. However, real‐time biofeedback and strategic modulation are crucial for adjusting customized clinical treatment adjustment. To achieve this purpose, we integrated illumination, temperature, and electromyographic (EMG) sensing elements into the optogenetic bioelectronic system to avoid overexposure caused by localized overheating and to provide functional recovery evaluation during neural regeneration, which guides the in situ adjustment of the intensity, frequency, and duration of illumination parameters controlled by a wireless connected programmable external control board. In this study, both in vitro and in vivo experiments were performed to examine the optical, thermal, and electrical characteristics of our bioelectronic system. On this basis, we demonstrated a series of standardized EMG results to evaluate the recovery condition and modify the illumination parameters of each test rat. Combining temperature monitoring feedback and EMG signaling feedback, our optogenetic bioelectronic system enables strategic optogenetic spinal cord injury (SCI) treatment through real‐time illumination modulation to achieve customized spinal cord injury treatment.

## Graphical abstract

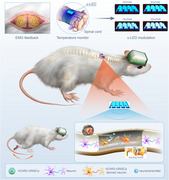

**Abbreviations**
μ‐LED: micro‐scale inorganic light‐emitting diode
Ach: acetylcholine
ADC: analog to digital converter
AFE: analog front end
BBB score: Basso, Beattie, and Bresnahan locomotor rating score
ECB: external control board
EMG: electromyographic
GAP 43: growth‐associated protein 43
GFAP: glial fibrillary acidic protein
hChR2: channelrhodopsin‐2
H‐E: hematoxylin‐eosin
MCU: main control unit
NF‐200: Neurofilament‐200
NSC: neural stem cell
PDMS: polydimethylsiloxane
PI: polyimide
PMMA: polymethyl methacrylate
PWM: pulse‐width modulation
SCI: spinal cord injury
SNSC: spinal cord‐derived neural stem cell
USB: universal serial bus
WB: Western blot

## 1 Introduction

More than 27 million patients worldwide have long‐term disability after spinal cord injury (SCI), either accidental trauma or related diseases [1, 2]. Nevertheless, SCI repair remains extremely difficult because of the limited regenerative potential of injured adult neurocytes, which obstructs the electrical transmission of neuronal activity and further hinders functional recovery [3, 4, 5, 6, 7]. In the past decade, tremendous efforts have been devoted to repairing SCI via neural stem cell (NSC) transplantation, which has already been shown to promote axonal regeneration [8, 9, 10, 11]. However, the differentiation results of transplanted NSCs are complicated and uncontrollable. Many types of NSC‐derived neurons with different neurotransmitters correspond to regulating different neural activities [12, 13]. Therefore, regulating the function of transplanted NSC‐derived neurocytes precisely during SCI regeneration would aid in reconstructing the neural circuit, which remains a significant challenge for SCI regeneration.

Optogenetics is a powerful technology that regulates neuronal activity by intervening in exogenously expressed light‐sensitive proteins triggered with specific wavelengths of light [14, 15]. Activating the light‐sensitive protein channelrhodopsin‐2 (hChR2) promotes the influx of cations, increasing the proliferation and differentiation of NSCs into neurons and astrocytes [16, 17]. Meanwhile, optogenetics allows precise activation or inhibition of neural activity of neurons [18, 19], making it the ideal candidate for regulating NSC‐derived neurocytes. In recent years, a series of implantable optogenetic bioelectronics have been developed (Table S1, Supporting Information), laying the solid foundation for developing optogenetic bioelectronic devices. However, current devices can only be fed with output signals that cannot provide biological feedback, let alone modulate the input signals based on feedback signals [20, 21, 22, 23, 24], which severely restrains the integration of diagnosis and treatment.

Recent advancements in optogenetic bioelectronic devices have resulted in integrating multifunctional systems [25, 26, 27, 28], which is more consistent with clinical practice. Based on the change of relevant indicators, the treatment plan can be adjusted accordingly. Herein, to avoid secondary damage to the injury site from the heat generated by excessive light exposure [29], we set the temperature sensor around the optic components. Moreover, we integrated the stimulation‐reception electromyographic (EMG) sensor [25] to provide real‐time SCI functional recovery feedback during SCI regeneration. Furthermore, we offer a customized regulation strategy to adjust input illumination signals based on relevant EMG feedback, achieving personalized optogenetic treatment for SCI.

**SCHEME 1:**
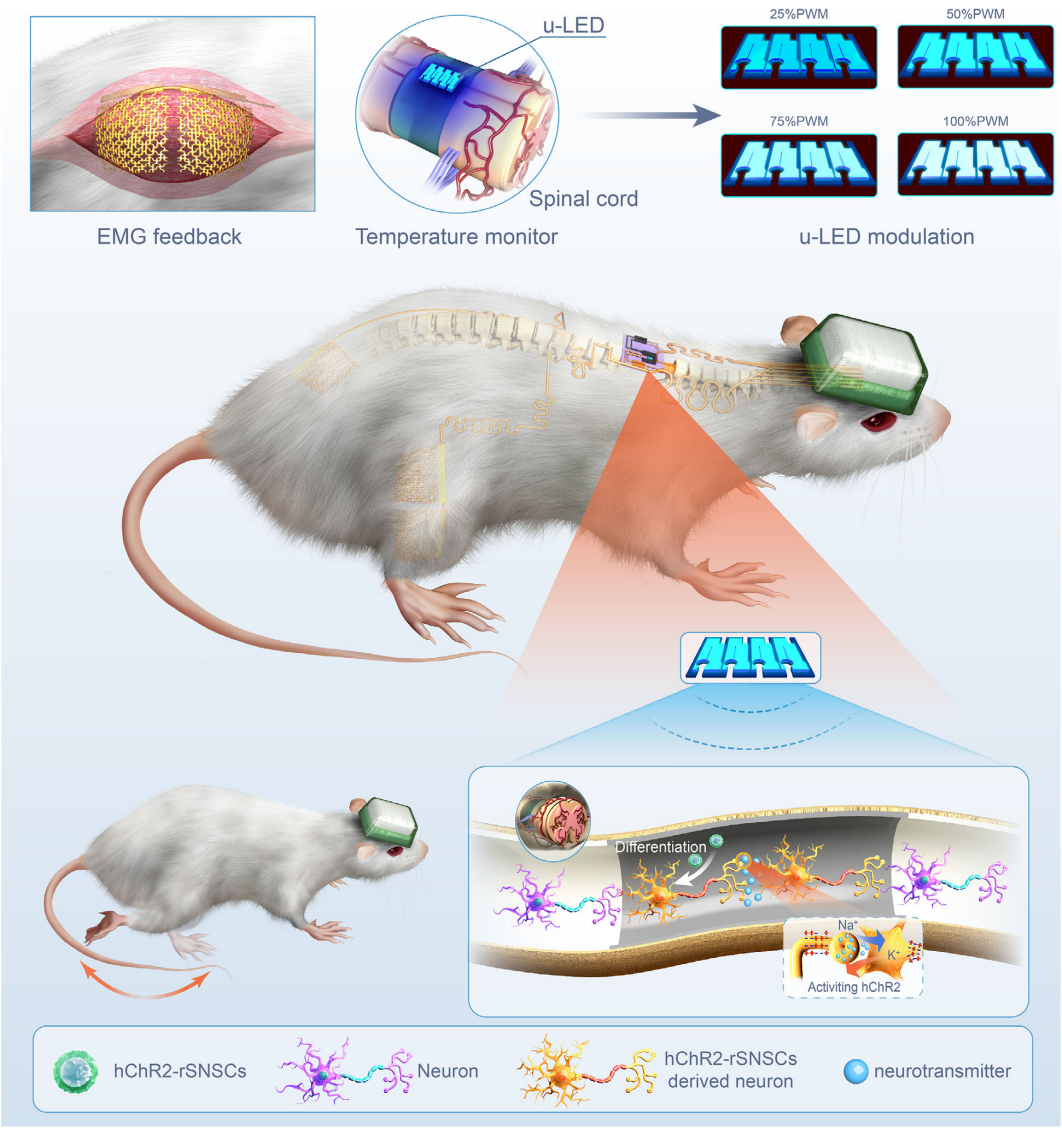
Real‐time feedback strategically regulates optoelectronics for customized optogenetic spinal cord regeneration. EMG, electromyographic; μ‐LED, micro‐scale inorganic light‐emitting diode; PWM: pulse‐width modulation; SNSC: spinal cord‐derived neural stem cell.

In the present study, we present soft, multimodal sensing and programmable optogenetic bioelectronics to regulate the behavior of differentiated NSCs and further assist the regeneration of neural function after SCI (Scheme 1). A series of in vitro and in vivo experiments is performed to examine the optical, thermal, and electrical characterizations of our optogenetic bioelectronic system, which not only verifies its flexibility and well integration with organisms but also confirms that the customized regulation strategy can effectively improve the recovery of SCI. To be more specifically, the optogenetic device developed here integrated the optical, thermal, and electrical components into one optogenetic bioelectronic system along with the Bluetooth‐connected input signal modulation capacity, including: (i) four micro‐scale 470 nm light‐emitting diodes (μ‐LEDs) that provide real‐time modulation of illumination intensity, duration, and frequency based on temperature monitor and EMG feedback to regulate the function of hChR2 transfected NSCs derived neurons, (ii) a temperature sensor that detect temperature change in situ to offer guide control of temperature stability during SCI regeneration through customized modulation of illumination, and (iii) two electrode arrays to provide intermittent and rhythmic electrical stimulation, and three electrode arrays to receive assessments of SCI functional recovery with real‐time EMG feedback through constant current electrical stimulation. Altogether, we offer a real‐time feedback regulation optogenetic bioelectronic system that provides personalized interventions for SCI regeneration.

## 2 Materials and Methods

### 2.1 Fabrication of Wirelessly Rechargeable Optogenetic Bioelectronic Device

Polymethyl methacrylate (PMMA) and polyimide (PI) substrates (10 µm) were prepared on a glass wafer. Sputtering titanium and gold (30 nm/200 nm) layers was used to graphically prepare the underlying circuit by photolithographic etching. A resistive temperature sensor was obtained by sputtering the titanium platinum layer (30 nm/150 nm) with ultrasonic‐assisted stripping. A Polydimethylsiloxane (PDMS) film (50 µm) with a square hole was laser‐cut and attached to the PI surface. A small amount of PI was poured into the hole and rotated evenly. PDMS was removed and heated at 90°C for 10 s on a heating plate, after which the μ‐LEDs were transferred to the exact position. The SU‐8 microneedle array was prepared by a specific process: sputtering copper (200 nm) spin‐coated photoresist (AZ5214E) and full exposure, spin‐coated the above photoresist again, patterned exposure, and appropriately extending the development time to form a depression zone, copper corrosion further into the depression zone to create a trapezoidal section structure more suitable for lift‐off three‐dimensional structure. Sputtering titanium and gold (30 nm/200 nm) layer for stripping; remove the remaining copper and photoresist. Encapsulation by Parylene coating; sputtered copper (200 nm) photolithographic etching, exposed the pad, microneedles, and the pores between them. RIE was performed with copper as a mask until the surface was removed along with the Parylene. Finally, the outline of the device was obtained by laser cutting, and the final device was obtained by penetrating the PI substrate with acetone and removing the PMMA (Figure S1, Supporting Information).

### 2.2 Characteristics of the Optical Component

Four μ‐LEDs (NSS‐W1025A5, Nationstar) with a size of 200 µm × 600 µm × 150 µm were thinned into a thickness of 8 µm. The volt‐ampere characteristic of the optical component was tested under the direct‐current power supply, and different duty cycles (25%, 50%, 75%, and 100% PWM) were tested under the control of the external control board (ECB) with or without stretching. With the control of different duty cycles, the relevant volt‐ampere value was recorded. Meanwhile, the package's reliability was examined in the saline solution at 37°C for 24 h under the control of the ECB.

### 2.3 Optical Simulation

The spherical crown model was adopted to simulate the μ‐LED light source to determine the surface optical power density, maximum effective depth, and area. The spinal cord was assumed to be a flat, uniform, ideal diffuser, and illuminated from one side with diffuse monochromatic light. The size of the μ‐LEDs array was 200 µm × 600 µm × 8 µm, the divergence angle was 154°, the height of the spherical crown was 150 µm, and the radius was 508 µm. Assuming conservation of energy, we calculated the geometric attenuation of intensity with distance from the μ‐LED surface *z* (*z* stands for the thickness of the sample), which can be expressed as:

$$\frac{I\left(z\right)}{I\left(z=0\right)}=\frac{\rho^{2}}{{\left(z+\rho \right)}^{2}}$$

The surface optical power density of the spherical crown is: (*P* stands for the optical power)

$$I\left(z=0\right)=\frac{P}{2\pi \rho H}$$

Considering the scattering and geometric loss, the full optical power density is: (*S* is the scattering coefficient per unit thickness, which is 10.3 mm^−1^)

$$\frac{I\left(z\right)}{I\left(z=0\right)}=\frac{\rho^{2}}{\left(Sz+1\right){\left(z+\rho \right)}^{2}}$$

Thus, the power density of light through the spinal cord thickness changes, and the formula is:

$$I\left(z\right)=\frac{\rho^{2}}{\left(Sz+1\right){\left(z+\rho \right)}^{2}}\frac{P}{2\pi \rho H}$$

Because the excitation opsin optical power must be greater than 1 mW mm^−2^, let *I*(*z*) be 1 mW mm^−2^ [30] calculate the effective range of μ‐LED irradiation *z*, and then calculate the area of the effective surface of μ‐LED irradiation A (Figure S4C, Supporting Information).

### 2.4 Temperature Sensor Simulation

The rat was ideally simplified into an oval shape of rat body trunk, with the spinal section containing the spinal cord, dura, muscles, and lamina; the optoelectronic device was simplified into a small area sandwich structure composed of the substrate, μ‐LED arrays, and the packaging layer; the specific size parameters are listed in Table S2, Supporting Information. The simulation model was modeled and assembled by Solidworks (Dassault Systemes) and imported to Abaqus 22020 (Dassault Systemes). A 10‐node, tetrahedral, secondary thermal flanges type (DC3D20) was used to mesh the rat body trunk, and a 20‐node, hexahedral, secondary thermal flanges type (DC3D20) was used to mesh the vertebral plate, spinal cord, μ‐LED arrays, substrate, and encapsulation layers. All simulation objects were considered isotropic mean values, and all materials corresponding to each part were assigned specific material attributes (Table S2, Supporting Information).

The simulation process was conducted at a constant temperature and humidity, the environmental boundary condition was set at 25°C, the convective heat transfer coefficient was 27 W m^2^ K^−4^, the average dimensionless body emissivity constant was 0.8, and the Stefan Boltzmann constant was 5.67 × 10^−8^ W m^2^ K^−4^. As the optoelectronic device was implanted in the rat for over 28 days, the body temperature was considered consistent, and the initial temperature was set at 37°C. The corresponding heating power was obtained by subtracting the output power from the luminous power of the thermal load. The corresponding heating power was calculated using the luminous power and voltammetry characteristic curves measured under different PWM waves to confer the thermal power load. The same 60 s luminous working time and 90 s cooling time as the test were adopted for simulation analysis.

### 2.5 Tensile Simulation Analysis of Serpentine Circuit

The μ‐LED arrays, temperature sensor, and electrode array in the device were simplified in the tensile simulation, and the external contour and internal metal wiring of the device were drawn in CAD2016. Abaqus 22020 (Dassault Systèmes) was imported to establish the 3D deformable shell. The structure grid was divided using the S4R cell type. Different areas were endowed with materials in the form of composite layers (Table S3, Supporting Information).

Static stretching mode was adopted, the simulation time was 1, the minimum time increment was 10^−8^, and the maximum time increment was 0.1. In the initial state, the linear segments at both ends of the serpentine circuit were fully constrained, and only the moving pairs in the tensile direction were released. A uniform load of 10^−6^ MPa, with minor disturbance, was applied to the upper and lower arcs of the serpentine circuit, and the disturbance was canceled after stretching. The displacement constraints of 38, 23, and 20 mm were applied to the straight‐line segment at the left end of the serpentine circuit.

### 2.6 Animal Model of SCI and Optogenetic Bioelectronic Device Implantation

All animal procedures were approved by the Institutional Animal Care and Use Committee of the Second Affiliated Hospital of Zhejiang University (AIRB‐2021‐1107). Twenty female rats (≈250 g) were used as the animal models for SCI modeling and the optogenetic bioelectronic device implantation. All rats were anesthetized with 1% (w/v) pentobarbital sodium (0.4 mL/100 g), shaved the head region, back region, buttock region, and right thigh region, then aseptically prepared with 70% ethanol and betadine solution. In preparing for spinal transection, the thoracic vertebra was exposed, the level of the T9‐T11 vertebral column was resected with laminectomy, and the complete transection was performed at the T10 segment. Precisely counted 10,000 hChR2‐rSNSCs were concentrated into 1 µL, then directly injected into the middle of the injury cavity; the direction and depth of injection were controlled by a specific limitator.

Once the hChR2‐rSNSCs were transplanted into the lesion site of SCI, the main part of the device, consisting of four μ‐LEDs and a temperature sensor, was also sutured to the paravertebral muscle, with μ‐LEDs located directly above the injury site of the spinal cord. Meanwhile, two stimulation electrode arrays were foisted along the gap between the lamina and placed right on the spinal cord. Then, a 2‐cm incision was incised above the rat skull, and the port for signal transmission and reception was glued and stitched to this area. The myoelectric detection electrode arrays were placed and sutured at the rectus femoris with the myoelectric ground electrode array at the gluteus maximus.

### 2.7 Immunofluorescence

For immunofluorescence detection, all samples were washed thrice with ddH_2_O, and their cell membranes were lysed with 0.3% Triton X‐100. The samples were incubated with 5% BSA at 37°C for 1 h and incubated overnight at 4°C with the appropriate primary antibody (anti‐neurofilament heavy polypeptide antibody, ab207176, Abcam; anti‐MAP2 antibody, ab11267, Abcam; anti‐choline acetyltransferase antibody, ab181023, Abcam; anti‐CD68 antibody, ab283654, Abcam; anti‐GFAP antibody, ab68428, Abcam; anti‐beta III Tubulin antibody, ab18207, Abcam; anti‐Ki67 antibody, ab16667, Abcam; anti‐GAPDH antibody, ab181602, Abcam), followed by the appropriate secondary antibody. The microspheres were finally observed under a fluorescence microscope. All antibodies used in the present study are listed in Table S4, Supporting Information.

### 2.8 Reinforcement Learning for Potential Optogenetic Treatment Strategy

**SCHEME 2:**
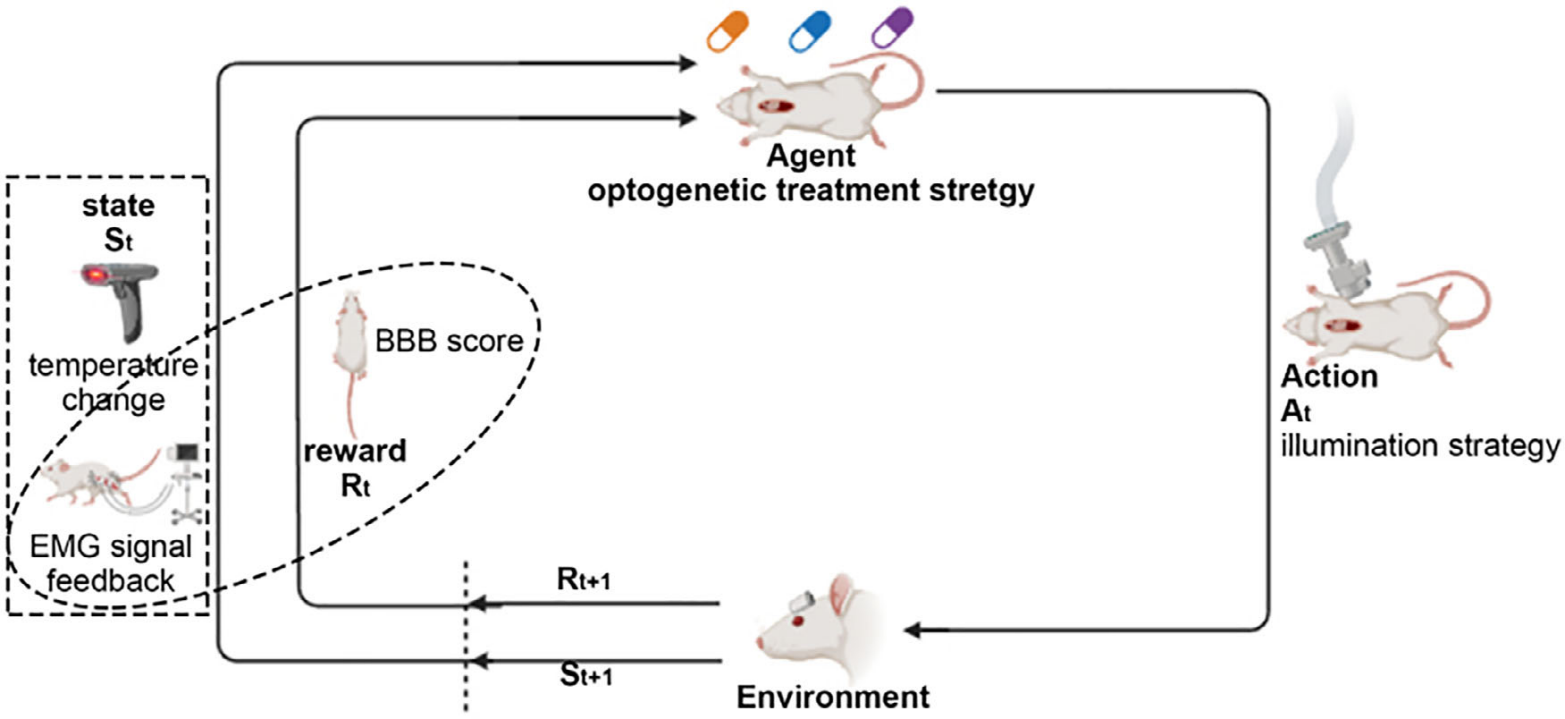
The agent‐environment interaction in reinforcement learning for potential optogenetic treatment strategy.

We formulate the learning problem as a reinforcement learning model for a Markov decision process (MDP) defined by the tuple M=⟨S,A,T,O,R,γ⟩, with S and A denoting state and action space, T (s'|s, a) the transition dynamics, R (s, a) the reward function, γ∈ [0, 1] the discount factor, and O the observation space. The framework is designed for both simulation and real‐world settings, transitioning from full observability in simulation (s ∈ S) to partial observability in the real world (o ∈ O). In this work, our goal is to customize the optogenetic treatment strategy for spinal cord injury with our bioelectronic system. The S state is the reflection of temperature change and EMG signal feedback, A action space is the illumination treatment strategy which determined by the Agent we are trying to achieve. The R reward function is defined based on the BBB score as well as the EMG signal feedback. This necessitates operating within a partially observable Markov decision process (POMDP), with the policy π (a|$o_{\leq t}$) mapping observations to action distributions to maximize the expected return (Scheme 2) $J=E\;\left[R_{t}\right]=E\left[\sum_{t}\gamma^{t}r_{t}\right]$.

### 2.9 Statistical Analyses

Data are expressed as mean ± standard deviation. All data was subject to outlier (ROUT method) and normality (Shapiro–Wilk) testing. Parametric and normal datasets were analyzed using the Student's *t*‐test and ANOVA test with post‐hoc Tukey's testing. All statistical analyses were performed using SPSS software (SPSS Inc., USA). The significance threshold was set at a *p*‐value < 0.05.

## 3 Results

### 3.1 Design of the Bioelectronic Complex

**FIGURE 1:**
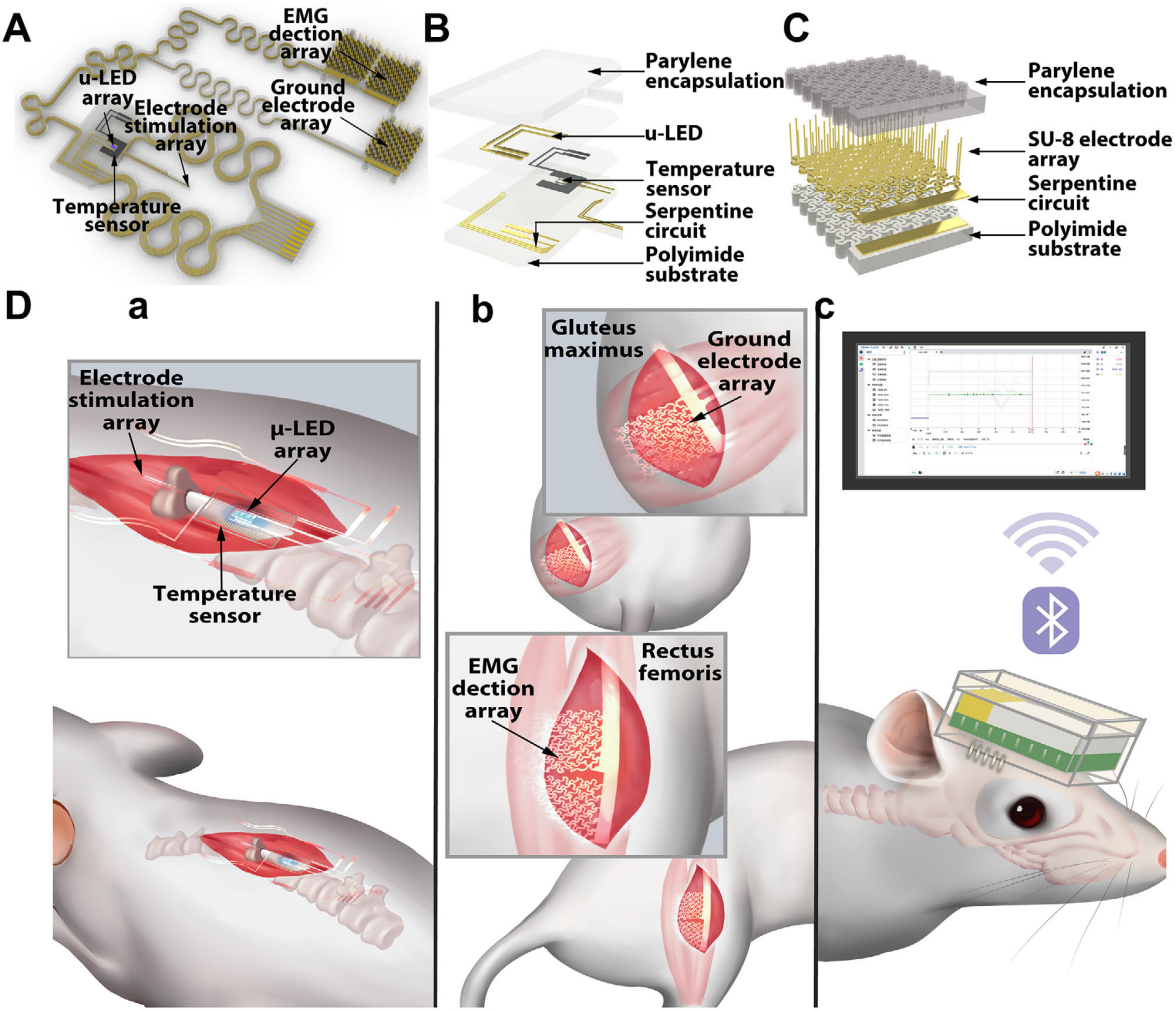
Soft, multimodal sensing and programmable miniaturized optoelectronic system for personalized optogenetic spinal cord regeneration. (A) Schematic diagram of the soft, multimodal sensing and programmable miniaturized optogenetic bioelectronic system. (B) Exploded view schematic diagram of the optical and thermal components. (C) Exploded view schematic diagram of the electrode array. (D) Schematic diagram of the implantation procedure and the operation principles of the optogenetic bioelectronic system.

To construct the feedback regulatory treatment mechanism in SCI regeneration, we propose a soft, multimodal sensing, and programmable miniaturized optogenetic bioelectronic system (Figure 1A). The whole optogenetic bioelectronic device (Figure S2, Supporting Information) consists of four parts: (i) optoelectronic μ‐LED arrays for photo‐stimulation, (ii) a temperature sensor for temperature monitoring, (iii) two EMG electrode arrays to provide intermittent and rhythmic electrical stimulation, and (iv) two EMG detection electrode arrays with a ground electrode array for EMG detection. Through the real‐time feedback of temperature and EMG, we can regulate the illumination parameter that could precisely control the activity of hChR2‐derived neurocytes. The detailed exploded schematic diagram of the illumination and temperature monitor component is depicted in Figure 1B, which is implanted right above the injury site of the spinal cord underneath the rat skin (Figure 1D(a)). Figure 1C shows the exploded schematic diagram of the electrode array, in which the ground electrode array is placed on the gluteus maximus, and the detective EMG electrode array is placed on the rectus femoris (Figure 1D(b)). As for the circuit connections between all four main functional components, the serpentine interconnects are used to accommodate the motion of the rats.

Furthermore, a custom‐designed ECB that controls the implanted optogenetic bioelectronic device was delicately developed and programmed (Figure S3, Supporting Information): the main control unit (MCU) integrated with the Bluetooth core is responsible for the operation logic of the entire device; the μ‐LED arrays that link to the analog front end (AFE), which is connected with the analog to digital converter (ADC), controls the input power of illumination, through controlling the pulse‐width modulation (PWM) wave duty ratio to achieve different input power of illumination; two electrode arrays, which are responsible for electrical stimulation, is controlled by the electrical stimulation module and connected with the MCU; the electrode array that links to the MCU is responsible to process the feedback EMG signals; the temperature sensor, which is also linked to the MCU, is responsible to receive temperature variation; the power supply unit is charged by a universal serial bus (USB) interface; finally, the whole board is powered by the 3.7 V ultrathin lithium battery (502030, Kexiangwei, China) and controlled by the computer through wireless connection.

The implantation and operational concepts of our soft, multimodal sensing, and programmable miniaturized optogenetic bioelectronic device system are presented in Figure 1D. Altogether, the illumination component was placed right above the spinal cord as the input signal, and the temperature sensor was placed around the μ‐LED arrays to monitor the temperature changes. Two electrode arrays were placed on the spinal cord to provide intermittent and rhythmic electrical stimulation; the other electrode arrays were placed on the rat's right leg to provide real‐time feedback of the rat's EMG after SCI, which acts as the output signals. All operations were controlled using the ECB and displayed through the custom‐designed computer program.

### 3.2 Optical Characterization of Optogenetic Bioelectronic System

**FIGURE 2:**
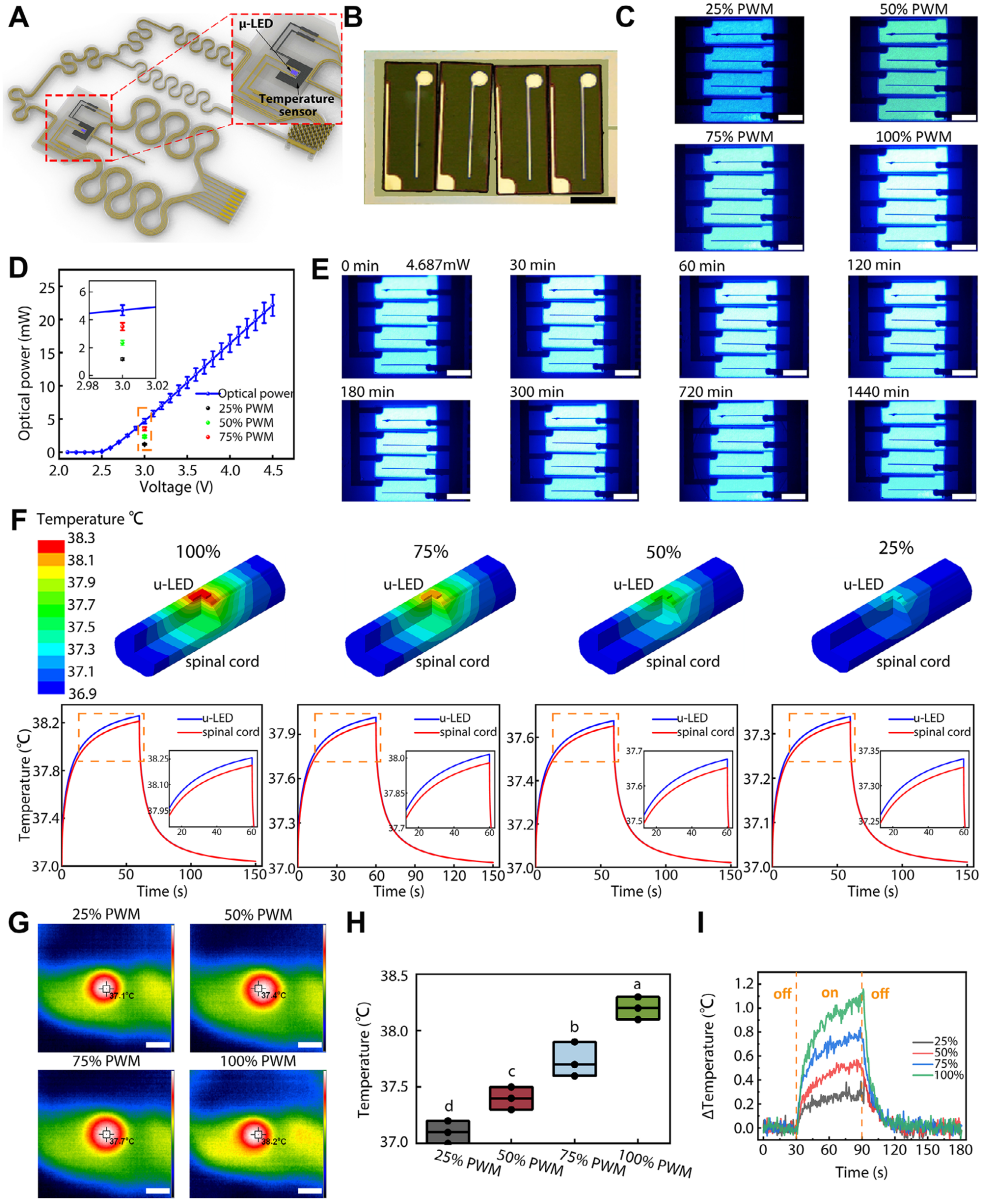
Optical and thermal characteristics of optogenetic bioelectronic system. (A) Schematic diagram of the optical and thermal components of the optogenetic bioelectronic system. (B) Microscope image of the μ‐LED arrays. Scale bar = 200 µm. (C) Microscope image of the μ‐LED arrays under the control of the duty ratio with different PWM waves. Scale bar = 200 µm. (D) Current–voltage characteristic of the μ‐LED arrays. (E) Long‐term stability of the μ‐LED arrays within the complex rat body fluid microenvironment simulation. Scale bar = 200 µm. (F) Thermal simulation of the in vivo μ‐LED arrays illumination temperature changes under the control of the duty ratio with different PWM waves. (G) Infrared temperature imaging results of the μ‐LED arrays illumination under the control of the duty ratio with different PWM waves. Scale bar = 1000 µm. (H) The quantitative statistics of (G), n = 3. (I) Realtime feedback of the temperature changes of the μ‐LED arrays illumination under the control of the duty ratio with different PWM waves.

It is essential to ensure the high quality, stability, and accuracy of the device components not only when tested in vitro but also when implanted and tested in vivo. Figure 2A depicts the zoomed schematic diagram illustrating the illumination component of the multifunctional optogenetic bioelectronic system. In detail, four 470 nm (Figure S4A, Supporting Information) μ‐LEDs with a size of 200 µm × 600 µm × 8 µm (Figure 2B) were arranged and transferred onto the PI substrate with four PWM waves (Figure 2C). The current–voltage characteristics of the μ‐LED arrays demonstrated that the turn‐on voltage was 2.5 V. The μ‐LEDs got cut off before the voltage reached 2.5 V, resulting in a linear trend of current–voltage after that (Figure S4B, Supporting Information). Under the control of the custom‐designed ECB, the μ‐LED arrays exhibited the same current–voltage characteristic. The luminous power reached 4.69 mW at the operating voltage (3 V). At the same voltage, the luminous power was also decreased proportionally by controlling the duty ratio with different PWM waves, which corresponds to 1.17, 2.34, and 3.52 mW, respectively, for 25%, 50%, and 75% PWM waves (Figure 2D). This allowed the real‐time modulation of μ‐LED illumination, which is the basis for personalized SCI treatment.

Through numerical calculation (Figure S4C, Supporting Information), we further examined the depth and optical power density of all four PWM waves. Under the control of a 75% PWM wave, the optical power density was 7.34 mW mm^−2^ on the surface of the spinal cord, and the power density decreased to 1 mW mm^−2^ at a depth of 236 µm. The maximum effective range of the μ‐LED arrays was 2.4 mm^2^ area with a depth of 236 µm. Also, under the control of 25%, 50%, and 100% PWM waves, the surface optical power densities achieved by μ‐LED irradiation were 2.45, 4.90, and 9.79 mW mm^−2^, respectively, and the maximum effective depth were 80, 170, and 289 µm, respectively, and the maximum effective surface area were 1.5 mm^2^, 2.00 mm^2^, and 2.76 mm^2^, respectively. The depth and optical power density of all four PWM waves were sufficient to enable the exponential regulation of hChR2 protein transfected NSCs.

Furthermore, the device was placed in the saline solution to further examine the long‐term stability of our optogenetic bioelectronic system within the complex rat body fluid microenvironment. Figure 2E shows that the μ‐LED arrays can stably and continuously work for 24 h in a humid microenvironment without being affected, satisfying the requirement of long‐term implantation. Herein, we demonstrated the high quality, stability, and accuracy of the optical component.

### 3.3 Thermal and Electrical Characteristics of Bioelectronic Systems

The temperature sensor is also installed around the μ‐LED arrays responsible for the temperature monitoring of the spinal cord during illumination to avoid secondary damage (Figure 2A). The sensor is calibrated in the working range of 31 to 43°C with a good linearity (Figure S5A,B, Supporting Information).

Based on the Pennes bioheat transfer equation, the simplified bioheat transfer equation and boundary condition equation are established without the blood mass perfusion heat transfer and metabolic heat production terms. The thermal simulation results indicate that both μ‐LED arrays and spinal cord temperature reached the highest point in 60 s. Even at 100% PWM wave input, the maximum spinal cord temperature only increased to 1.213°C, far less than the biocompatibility requirement of 2°C temperature change [22] (Figure 2F). After 90 s, the heat gradually spread out, and the temperature of the μ‐LED arrays returned. Figure 2G shows the real in vivo infrared imaging of the temperature sensor after implantation. The 100% PWM wave of μ‐LED arrays caused a temperature change of 1.2°C ± 0.08°C, which is also within the safe range of the biocompatibility requirement (Figure 2H). Under the control of the ECB, the thermal sensor in vivo exhibited similar results to the simulation (Figure 2I). Altogether, we demonstrated the relationship between the illumination and the temperature changes, which provides the boundary reference for regulating specific optical strategies. The integration of temperature sensors in our optogenetic device has proven its efficacy and safety in preventing secondary damage during the illumination of SCI regeneration.

**FIGURE 3:**
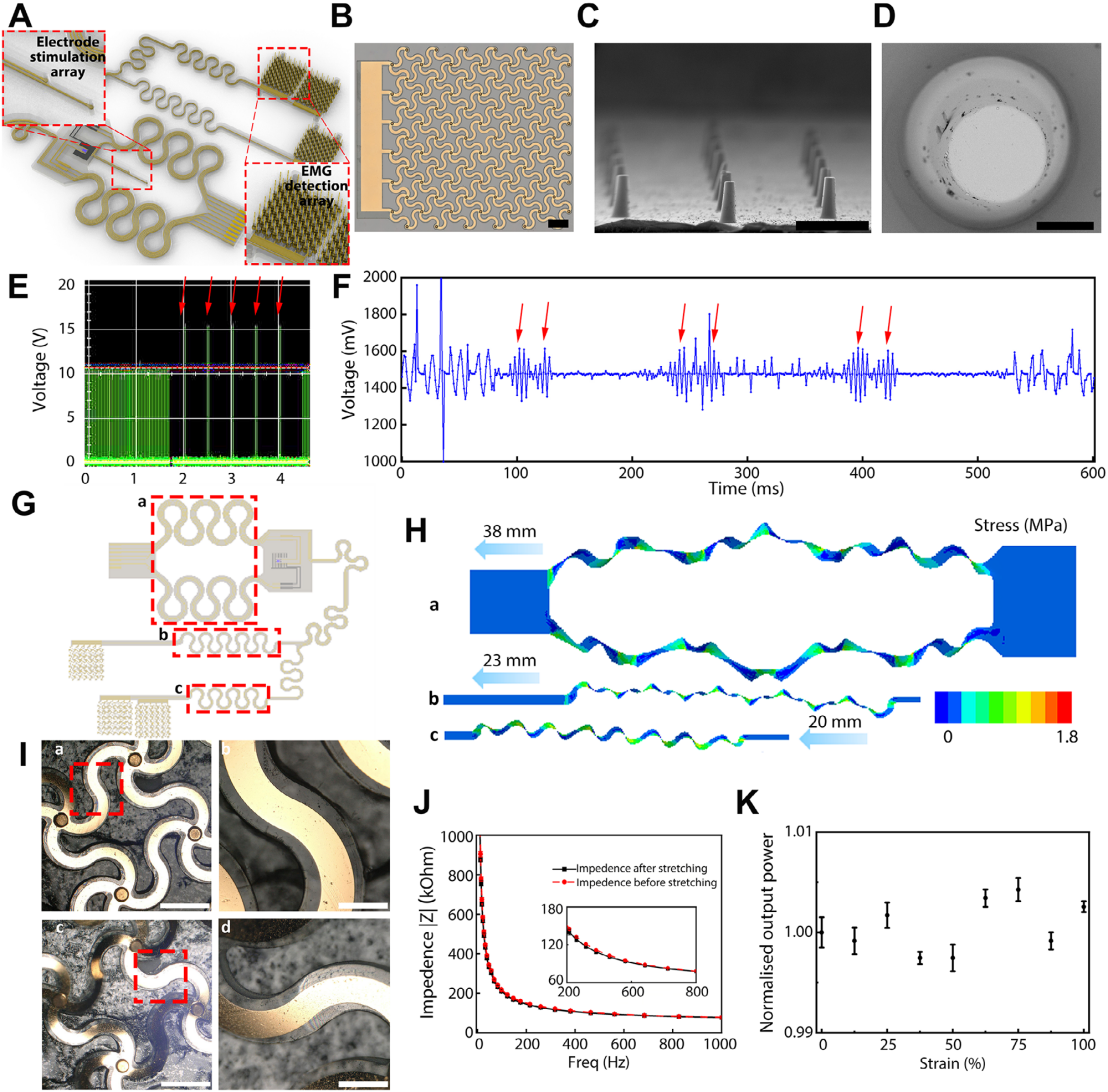
Electrical and stretch characteristics of optogenetic bioelectronic system. (A) Schematic diagram of the electrode array components of the optogenetic bioelectronic system. (B) Actual image of the electrode array. Scale bar = 1000 µm. (C,D) SEM results of the electrode array. Scale bar = 200 µm. (E) Pattern of the intermittent and rhythmic electrical stimulation. (F) Representative EMG results of the sham‐operated rat. (G) Schematic diagram of the serpentine circuit of the optogenetic bioelectronic system. (H) Simulation results of stress and strain values of the serpentine circuits. “a, b, c” is corresponding to “a, b, c” in Figure 3G. (I) Actual image of the electrode array before and after stretching. Scale bar = 500 µm. (J) Loading capacity of the serpentine circuit in electrode array during 1000 fatigue cycle tests. (K) Normalized input power of the μ‐LED arrays during the stretch, n = 3.

Figure 3A is the representative schematic diagram of the electrode array, including both stimulation and reception parts. Figure 3B depicts the actual light microscope result of the electrode array. The detailed morphology of the electrode array needle with scanning electron microscope (SIGMA 500, Zeiss) is shown in Figure 3C,D, along with the light microscope result (Figure S6, Supporting Information). Furthermore, we patterned the electrode array as serpentine style to meet the deformation during the movement of rats. The electrode shape was conical, which can make a better connection with the muscles. As to the functional tests, the pattern of the intermittent and rhythmic electrical stimulation is presented in Figure 3E, and the EMG receiving function of the electrode arrays was tested with a sham‐operated rat with the implantation of our optogenetic bioelectronic system (Figure 3F).

Herein, we provide a soft, multimodal sensing, miniaturized optogenetic bioelectronic system with high quality and strong stability to provide stable 470 nm illumination, accurate temperature monitoring as well as quantitative EMG detection.

### 3.4 Effect of Stretch Characteristics on Optical and Electrical Signals

The serpentine interconnect that connects all the functional components could perfectly adapt to the deformation caused by rat activity and growth (Figure 3G). However, the stretching deformation of the circuit might lead to the circuit aging and softening crack damage, thereby affecting the operation of the functional components.

First, the stretch characteristic of the circuit was examined to meet the rat's physiological environment. According to the simulation results, the maximum stress and strain points were inside the circular arc on both sides of the serpentine wire, and the simulated strain value and elongation were all within the tolerable range of the material, which was 38 mm (145.04%), 23 mm (92.00%), and 20 mm (99.01%) (Figure 3H). The maximum stress values of the three lines from top to bottom were 50.49 MPa, 54.64 MPa and 55.14 MPa, and the strain values were 1.59%, 1.72% and 1.59%, respectively. The actual stretching results of each group of the serpentine circuit were 40 mm (152.67%), 24 mm (120.00%), and 21 mm (103.96%), which were similar to the simulation results (Figure S7, Supporting Information). In conclusion, the serpentine circuit can achieve a total elongation of 85 mm, which certainly resolves the stretching requirement of the rat's activity and growth.

The relative effect of stretch on optical and electrical signals was then tested to ensure the stability of the optogenetic bioelectronic system. The serpentine circuit involved in the electrode array not only enabled the electrode array to better fit with the irregular surface of the muscle but also provided certain ductility in the process of rat activity (Figure 3I). Meanwhile, the impedance of the electrode array remained the same with or without stretching (Figure 3J), demonstrating the strong stability of our electrode array after implantation. Finally, the normalized input power of the μ‐LED arrays shows relatively stable results during the stretching, further ensuring the high quality and stability of the optogenetic bioelectronic system (Figure 3K).

### 3.5 Evaluation of the Bioelectronic Device in Rat SCI Model

To further evaluate whether our bioelectronic device can functionally control the intensity, duration, and frequency of illumination and provide real‐time feedback of temperature and quantitive EMG results in freely moving rats, we conducted optogenetic experiments by transfecting AAV9‐hSyn‐hChR2(H134R)‐EYFP (26973, Addgene) into Sprague‐Dawley (SD) rat spinal cord‐derived NSCs (rSNSCs), which were isolated and preserved for in vitro experiments (Figure S8, Supporting Information) and in vivo transplantation. The in vitro result showed promising neuron differentiation capacity and is capable of differentiation into acetylcholine neurons (Figure S8A, Supporting Information). The hChR2‐rSNSCs exhibited promising neurocyte differentiation capacity in vitro [16, 17], with 470 nm illumination 60 s thrice per day at 50% PWM, seems to be suitable for the in vivo experiment as a start (Figure S8B–D, Supporting Information).

**FIGURE 4:**
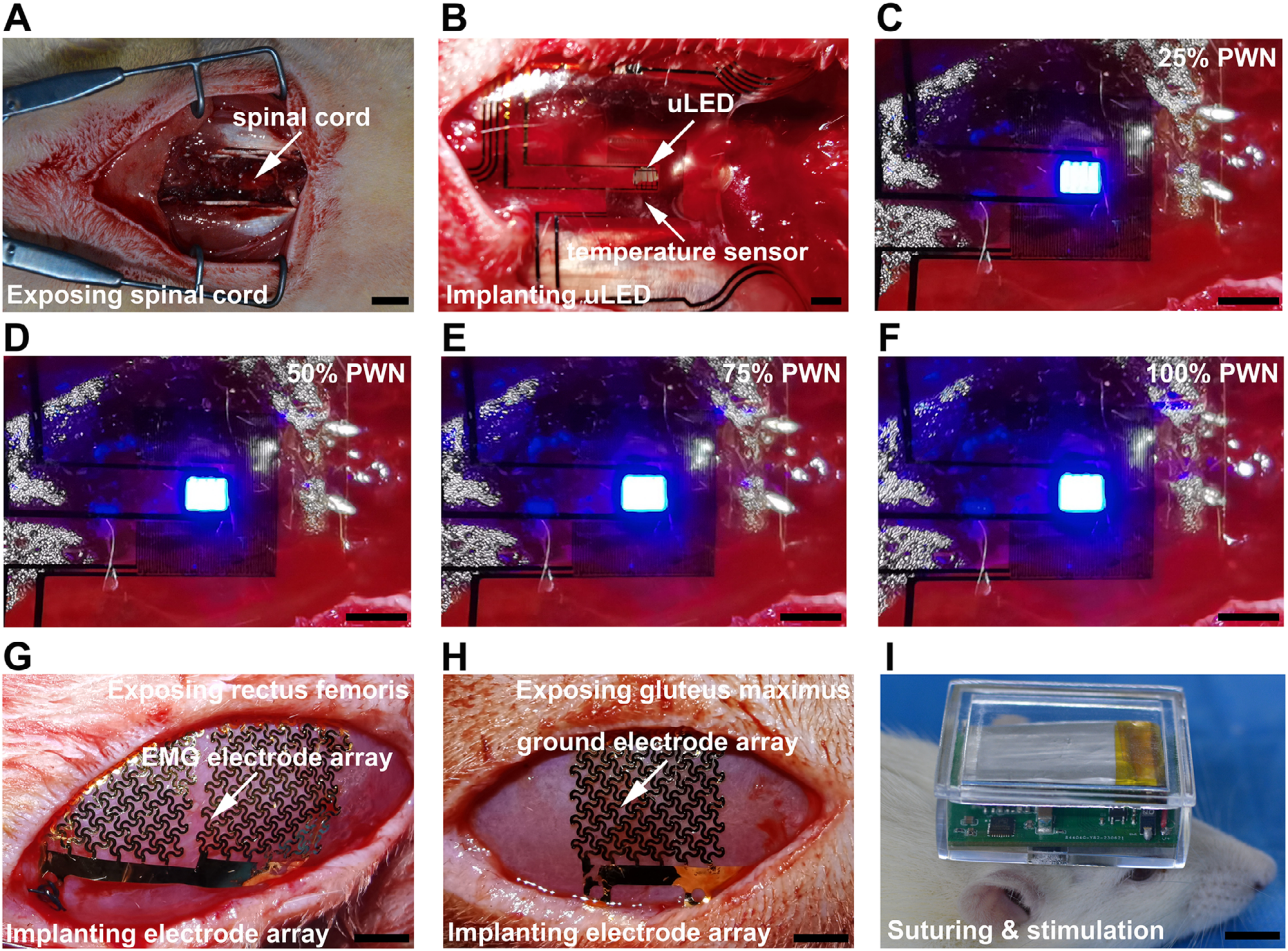
Surgical implantation, operation, and acute demonstration of optogenetic bioelectronic system. (A) Surgical procedure for exposing spinal cord. Scale bar = 1000 µm. (B) Implantation of the μ‐LED arrays and temperature sensor components. Scale bar = 1000 µm. (C–F) Actual image of the μ‐LED arrays under the control of 25%, 50%, 75%, and 100% PWM. Scale bar = 1000 µm. (G) Implantation of the EMG electrode array onto the right rectus femoris. Scale bar = 1000 µm. (H) Implantation of the ground electrode array onto the gluteus maximus. Scale bar = 1000 µm. (I) Suturing and stimulation of the optogenetic bioelectronic system. Scale bar = 1000 µm.

For the implantation of the device (Figure 4), a 2‐cm incision was incised above the rat skull, and the port for signal transmission and reception was glued and stitched to this area. Once the hChR2‐rSNSCs were transplanted into the lesion site of SCI, the main part of the device, consisting of four μ‐LEDs and a temperature sensor, was also sutured to the paravertebral muscle, with the μ‐LEDs located directly above the injury site of the spinal cord and two stimulation electrode arrays were foisted along the gap between the lamina and placed right on the spinal cord. The myoelectric detection electrode arrays were placed and sutured at the rectus femoris with the myoelectric ground electrode array at the gluteus maximus (Figure S9, Supporting Information).

After analyzing the EMG signal statistics of ten SCI SD rats transplanted with 10,000 hChR2‐rSNSCs [3, 31] and implanted with the optogenetic bioelectronic system, and illuminated thrice for 60 s each day. Through feedback signal collection, we initially obtained a series of typical EMG results with the best recovery condition and set it as the standardized EMG results for SCI regeneration to maximum avoid the individual difference between each rat (Figure S10, Supporting Information). Afterward, all three tested rats were treated under the same conditions, but with a customized modification of the illumination strategy based on EMG feedback every week, trying to meet the best recovery condition of each rat at the same level.

**FIGURE 5:**
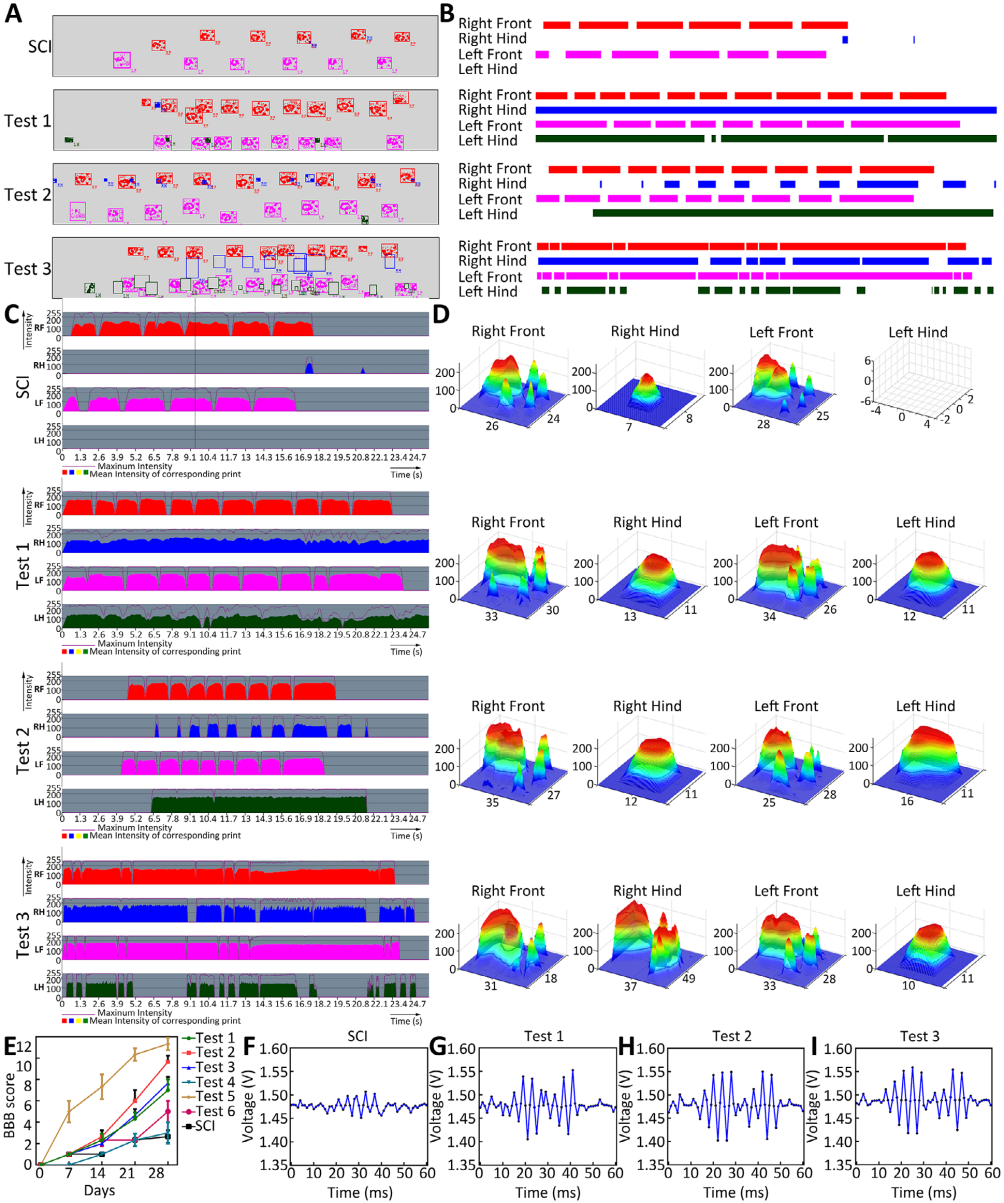
Functional behavior evaluation of rat SCI model. (A) Footprint images of the rat SCI models. (B) Footprint analysis of the SCI rats’ limb movement. (C) Footprint pressure analysis of the SCI rats’ limb movement. (D) Representative 3D footprint pressure images of the SCI rats’ limb movement. (E) BBB score of all tested rats’ recovery status in four weeks, n = 3. (F–H) Representative EMG results of three test rats after 4 weeks of regeneration compared with the SCI group.

**FIGURE 6:**
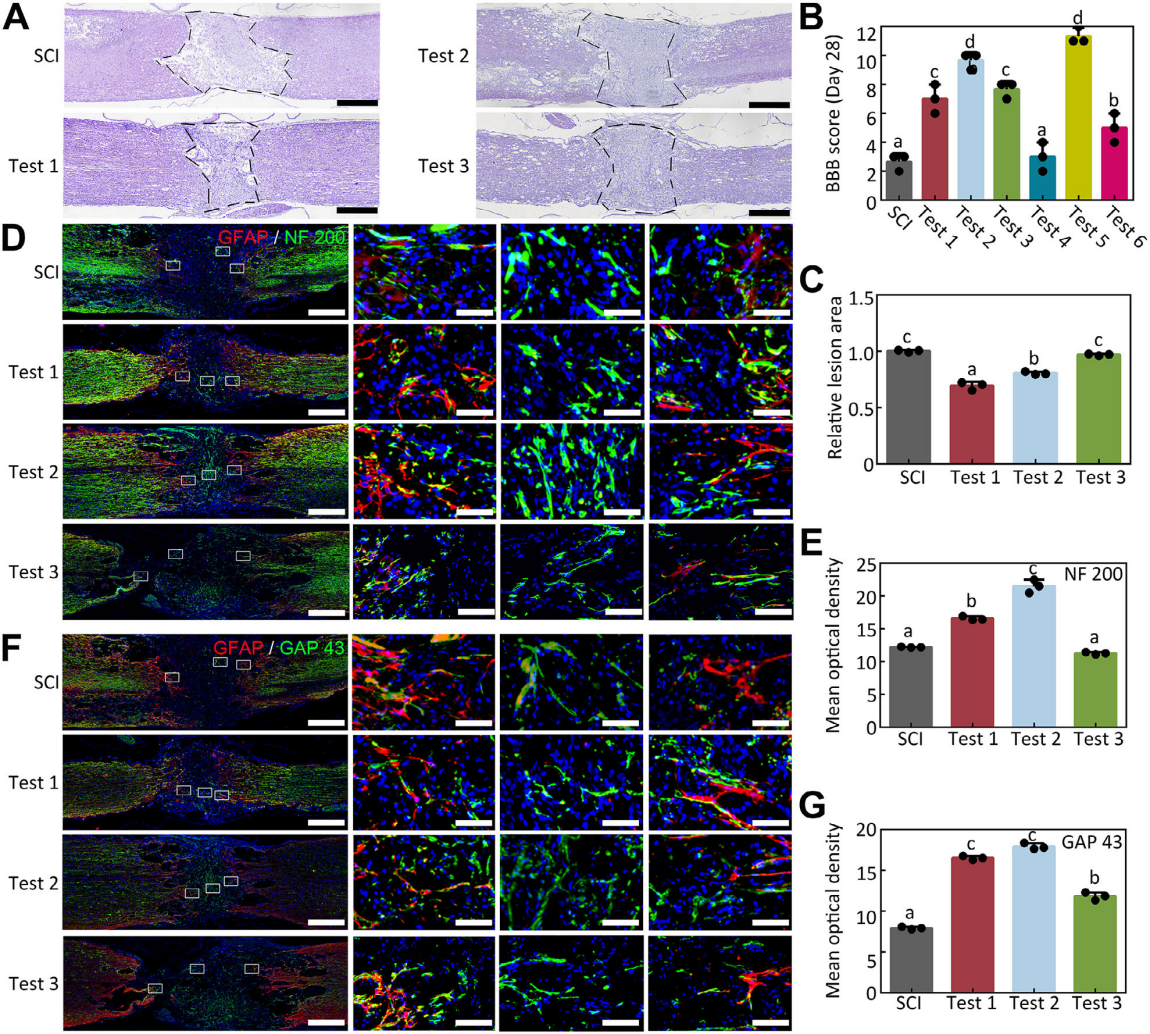
Histological evaluation of the regeneration in the rat SCI model. (A) H–E staining results of the regeneration in the rat SCI model after 4 weeks. Scale bar = 1000 µm. (B) BBB score of the rats’ recovery status on day 28, n = 3. (C) Relative lesion area of all three rats after 28 days regeneration, n = 3. (D) Representative NF 200 and GFAP IF results of all three rats after 28 days regeneration. Scale bar = 1000 µm. (E) The quantitative statistics of (F), n = 3. (F) Representative GAP 43 and GFAP IF results of all three rats after 28 days regeneration. Scale bar = 1000 µm. (G) The quantitative statistics of (G), n = 3.

After 28 days of recovery and observation, the motor function was examined using the kinematic footprint pose analysis system (RD1128‐FP, Shanghai Mobile Datum Information Technology Co., Ltd.). The representative footprints of the tested rats are shown in Figure 5A, and their motion frequencies of both front and hind limbs are presented in Figure 5B. All four limbs of each rat's footprint pressure in a motion cycle are shown in Figure 5C, and the representative 3D footprint of each limb is presented in Figure 5D. In an equal period, all three rats produced relatively different results in the recovery of motor function. Test rat 2 exhibited a relatively better trend of motor functional recovery than Test rats 1 and 3, which were relatively similar. The Basso, Beattie, and Bresnahan locomotor rating score (BBB score) indicated similar results (Figures 5E and 6B). The representative EMG results of each rat at 28 days are presented in Figure 5F–I.

### 3.6 Histological Evaluation of the Regeneration in Rat SCI Model

In this study, we presented a customized SCI regeneration strategy, which integrated the diagnosis and treatment. With the feedback of EMG and temperature changes, we evaluated the recovery of neural function after SCI within the safe temperature range. By controlling the ECB, we could regulate the intensity, frequency, and time of the illumination, optimize the optical scheme, and finally achieve the optimal SCI treatment strategy.

To prove our hypothesis, we sacrificed all the tested rats, and their spinal cords were embedded in paraffin and sectioned (4 µm). The hematoxylin–eosin (H–E) staining results (Figure 6A) revealed the histological morphology of the injured spinal cord on day 28 after SCI. Severe injury was observed in the longitudinal section of all three rats’ spinal cord injury sites with apparent cavity formation. The relative injury area of the spinal cord in tested rat 2 was slightly smaller than that in the other two rats, and there was less chaotic tissue at the injury site (Figure 6C). Meanwhile, amplified H–E result showed that there was a similar degree of chronic inflammatory infiltration in both the SCI group and the Test group. Also, the biocompatibility results of one month after implantation showed no obvious abnormalities in the H&E staining of heart, liver, spleen, lung, and kidney tissue sections (Figure S11, Supporting Information).

Immunofluorescence (IF) was used to analyze the histological regeneration results after SCI on day 28. All the blue light is stained with DAPI to reflect the nucleus of the cells. Neurofilament‐200 (NF 200) protein is the intermediate filament protein in neurons. Figure 6D shows the typical NF 200 positive neuron filament elongation in the form of dots or tubules at the injury site of all three rats. The quantitative result showed that more neurofilament regenerated through the lesion area of Rat 2, while a small number of regenerated neurofilament were observed in rats 1 and 3 (Figure 6E). Another neuron protein, growth associated protein 43 (GAP43), a nerve cell membrane protein, which participates in the development and plasticity of nerve cells, was examined to further prove the regeneration after SCI. All three rats exhibited representative green tubular GAP 43 protein fluorescence at the injury site of SCI; there were positive GAP 43 axons breaking through the glial fibrillary acidic protein (GFAP) glial scar boundary on both sides of the injury edge (Figure 6F). The quantitative result showed that neonatal axons were observed in all Test groups, with less in rat 3 (Figure 6G).

**FIGURE 7:**
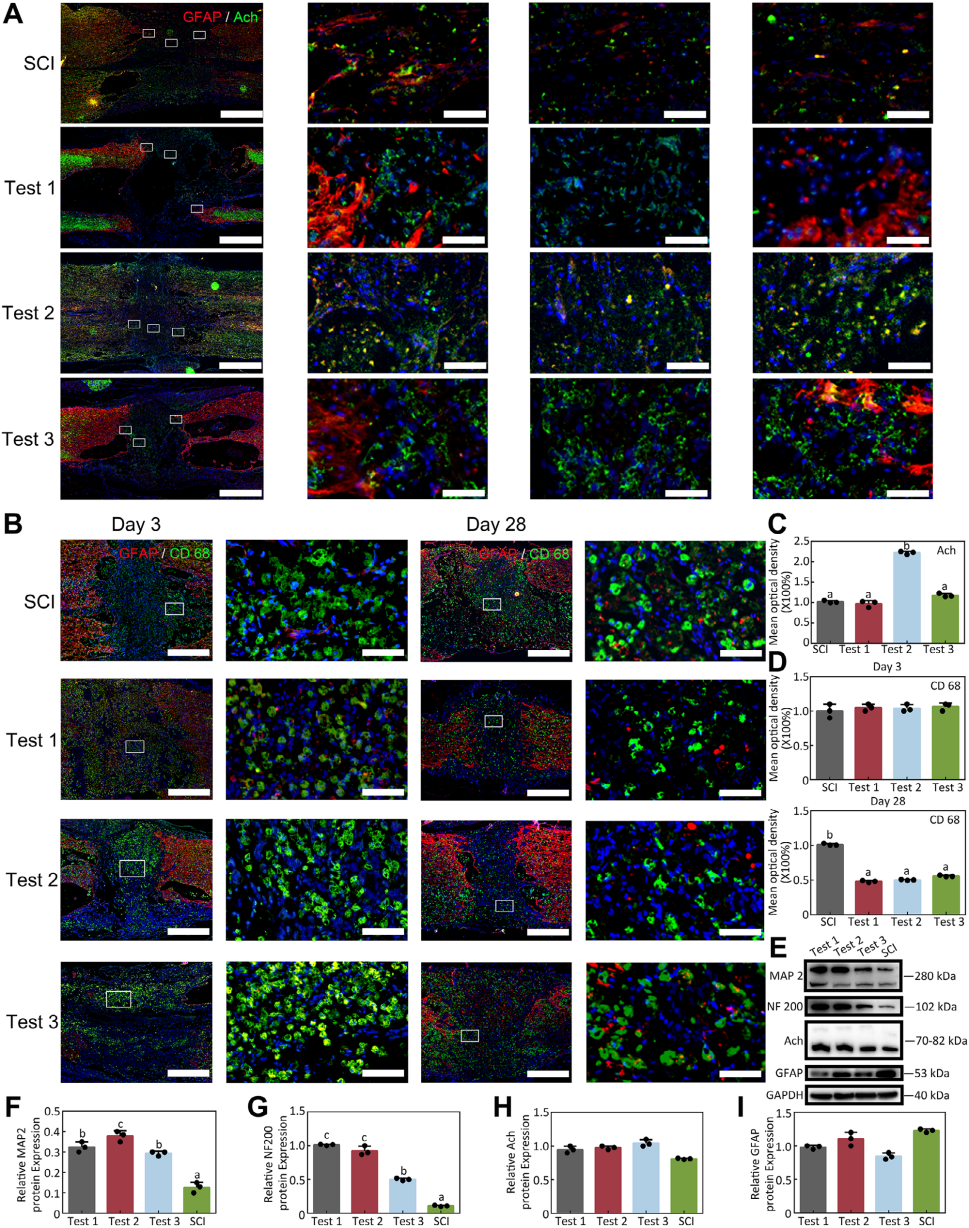
Histological function evaluation of the regeneration in the rat SCI model. (A) Representative Ach and GFAP IF results of all three rats after 28 days regeneration. Scale bar = 1000 µm. (B) Representative CD 68 and GFAP IF results of all three rats after 3 days and 28 days regeneration. Scale bar = 1000 µm. (C) The quantitative statistics of (A), n = 3. (D) The quantitative statistics of (B), n = 3. (E) Representative protein expression analysis of all three rats after 28 days regeneration. Scale bar = 1000 µm. (F–I) The quantitative statistics of (E), n = 3.

Moreover, acetylcholine (Ach) neurons, one of the main functional neurocytes, were examined, as shown in Figure 7A. Typical Ach proteins were observed in all three rats, with Rat 2 observed the most (Figure 7C). The western blot (WB) results shared similar conclusions (Figure 7E–I). However, Rat 2 showed relatively better neural recovery than the other two rats, consistent with the motor footprint analysis, BBB score, and EMG results. Furthermore, the inflammatory infiltration, probably caused by the optogenetic bioelectronic system implantation, is observed in Figure 7B,D, which makes no difference in all three rats. Although it seems that the inflammatory infiltration did not affect the result of neuron function regeneration following the implantation of the optogenetic bioelectronic device, this raises new questions for future research.

## 4 Discussion

We presented a soft, multimodal sensing, and programmable optogenetic bioelectronic system that integrated the illumination modulation with a temperature monitor and quantitative EMG detection feedback. This system allowed possible application for real‐time modulation and feedback during the photo‐activation of transplanted hChR2‐NSCs to provide personalized SCI treatment. Herein, the temperature sensor was used to avoid secondary damage because of excessive illumination; the temperature changes and EMG results were analyzed to personalize the illumination frequency, intensity, and duration for each rat. Our real‐time feedback system regulates the light intensity through evaluating temperature change and EMG results. During the process of light stimulation, the temperature sensor monitors in real time to prevent excessive light exposure that causes overheating. In the meantime, EMG results were obtained from the rat's rectus femoris, through comparing with the standard recovery EMG results at different times (Figure S10, Supporting Information), we were able to turn up the light intensity if there is a significant gap. Our final goal is for the results of each experimental group to converge toward our standardized EMG results.

The difficulties of combining optical, thermal, and electrical within a miniature and biocompatible platform system include controlling the thickness and radiation depth of the μ‐LED array. Avoiding the temperature sensor from being affected by the operation of the micro‐LED array. Collecting electromyographic signals with microneedle electrode arrays from the rectus femoris muscle of experimental rats without hurting the muscles. The serpentine circuits of flexible electronic devices ensure the stability of the entire system in rats while connecting it. Last but not least, the ECB is burned to control the input and output of the integrated systems. Our research would accelerate neuroscience research through robust real‐time modulation and feedback in any in vivo models, thereby contributing to the experimental investigation of neural functions and treatment (See Scheme 1 and 2).

Although our optogenetic bioelectronic system shows novel features, its design can be improved. The device was supported by an ultra‐thin, stretchable PDMS membrane (13.46 ± 0.25 µm), allowing subdermal implantation in rats. The serpentine alignment applied throughout this device's structure further ensures that its functionalities will remain relatively stable despite the growth and movement of rats [32, 33]. However, the current design cannot integrate the whole optogenetic bioelectronic system into a rat, primarily because of the bulkiness of the integrated chip code and the capacity of the lithium battery. Wireless Bluetooth technology and an ultra‐thin, flexible battery with sufficient capacity as replacements for the ECB would further scale down its size without compromising its functionality, making fully integrated implantation possible.

The design and manufacture of the serpentine circuit are also crucial [34, 35, 36]. Despite the growth and movement of the model rats, it is essential to ensure that the optical, thermal, and electronic components are in place. The main functional parts were sutured onto the relevant muscles, and the rest of the connecting circuits were shaped into serpentine to meet the requirement of stretching and folding. Our results demonstrated that the serpentine circuit structure ensured the stability of the device's function against the motion of the rats. Meanwhile, with the stretching and folding of the connecting serpentine circuit, the optical, thermal, and electronic components remained stable, further meeting the deformation requirements generated during the movement of rats without affecting the main components of the optogenetic bioelectronic system.

There is still much room for improvement in our optogenetic system for further clinical translation. The size and thickness of the flexible electronic device can be reduced or increased to match the size and physiological environment of other animals or even the human body [37, 38]. The difficulty of this work lies not in increasing but in reducing the size of the device, which seems promising for further clinical translation. Meanwhile, we choose an ultrathin lithium battery to supply the ECB, which makes it easy for the wireless ECB to recharge its power. Of course, we plan to customize ultra‐thin solar cells as a future improvement direction, which will not only be thinner and lighter but also more convenient.

One more point must be mentioned, inflammation is quite important in SCI regeneration. Previous literature indicates that inflammation will not only lead to the formation of cystic cavities wrapped by scar tissue, but also seriously hinders axon regeneration [39, 40]. Based on this research, we propose two possible directions. First, by altering the wavelength of the u‐LED array, anti‐inflammatory treatment can be carried out on the site of SCI through red light irradiation [41, 42]. Second, anti‐inflammatory drug delivery can be achieved through adding microneedle components [20]. These are all parts that can be improved on this device. We believe this can better help the clinical translation of our optogenetic system.

The EMG results are crucial in SCI functional regeneration evaluation. However, it is almost impossible to quantify the changes in EMG signals after SCI. Therefore, to receive EMG feedback from the rectus femoris of the tested rats, we provided stable intermittent transverse piezoelectric stimulation at the upper end of the spinal cord area. Herein, after the transplantation of hChR2‐rSNSCs and the implantation of the soft, multimodal sensing, and programmable optoelectronic system, we summarize the representative EMG changes of SCI rats within 28 days and propose a rather plain strategy for optogenetic regulation. The standardized EMG results are presented based on the illumination condition performed in vitro, and increase the PWM wave when the recovery condition of the test rats does not reach the standard, but with a limitation, which is quite consistent with the clinical principle. Of course, this strategy cannot satisfy the needs of optogenetic SCI treatment. With the advances in artificial intelligence, deep learning, collecting, and analyzing large numbers of clinical data has become increasingly common, influencing the direction of further bioengineering research. Therefore, we plan to further improve our bioelectronic device and collect EMG and temperature feedback results with a more detailed schedule during the regeneration of SCI. Ultimately, we would continue using deep learning for systematic analysis and provide detailed and reliable guidelines for applying optogenetic bioelectronic systems in clinical SCI treatment.

Last but not least, we selected AAV as the viral vector and transfected hChR2 into rSNSCs, which has been widely applied in optogenetic research [43, 44, 45]. However, although AAV is considered to have extremely high biological safety, it is still believed to have potential carcinogenicity [46] and liver toxicity [47, 48], which has a certain impact on the clinical translation prospects of this subject. Recent research showed that transcription factor A mitochondrial (TFAM)–based polyplex (TFAMoplex) [49, 50], fusogenic and safe transfection‐proteolipid vehicle (FAST‐PLV) [51], and precise RNA‐mediated insertion of transgenes (PRINT) [52] seem to be the possible solution. With the development of technology, safe hChR2 protein transfection can surely provide a good foundation for the clinical translation of this study.

In this study, our optogenetic bioelectronic devices achieved the functional integration of optical, thermal, and electrical signals, allowing the intervention and feedback approach to formulate personalized treatment during SCI regeneration. One of the input signals controlled the illumination, including the intensity, frequency, and duration, which plays an important role in hChR2‐NSC differentiation and the depolarization of hChR2‐NSC‐derived neurocytes. The other two output signals were designed to receive the temperature changes and EMG feedback. The temperature sensor was placed to monitor the temperature variation around the injury site of SCI, which prevents secondary damage caused by excessive illumination. The EMG electrode and transducer provided quantitative neuro‐electrophysiological feedback, directly reflecting the motor functional recovery of SCI rats. Both temperature monitoring and EMG feedback are essential for the schedule of personalized optogenetic modulation in SCI treatment. Our multimodal sensing optogenetic bioelectronic system provided a promising clinical translation strategy for future nerve injury regeneration. Furthermore, our integrated real‐time feedback regulation devices can be applied in various implantable devices, such as cardiac pacemakers, gastric pacemakers, and urinary bladder detectors. Despite the good organization of our optogenetic bioelectronic device, the transplantation of light‐sensitive protein‐transfected NSCs remains a serious ethical issue, which requires further research to overcome the genetic safety of optogenetics [53, 54].

## 5 Conclusion

Our multimodal sensing optogenetic bioelectronic system demonstrated its ability to regulate the behavior of differentiated NSCs and aid in the regeneration of neural function after SCI. Unlike conventional optogenetic bioelectronic devices, most of which have focused on the input of illumination to activate light‐sensitive proteins, we offer real‐time illumination modulation based on temperature monitoring and integrated electrode stimulation, along with EMG feedback that reflects the process of SCI regeneration. Herein, we present a soft, multimodal sensing, and programmable miniaturized real‐time feedback regulation optogenetic bioelectronic system for the regeneration of SCI, which provides a platform for further clinical translation in nerve injury treatment.

## Author Contributions

Kaishun Xia, Qi‐Xin Chen, Ying Chen, and Cheng‐Zhen Liang designed experiments. Kai‐Shun Xia, Jiangjie Chen, Jingkai Wang, Yuang Zhang, Kesi Shi, Yi Li, and Chenggui Wang carried out experiments. Kai‐Shun Xia, Hao Li, Yiqing Tao, and Xinmao You analyzed experimental results. Kai‐Shun Xia wrote the manuscript. Hao Li, Ying Chen, Fangcai Li, and Qixin Chen helped to polish this manuscript. All authors critically read and revised the manuscript and approved its submission for publication.

## Conflicts of Interest

The authors declare no conflicts of interest.

## Supporting information




**Supporting File 1**: exp270159‐sup‐0001‐SuppMat.docx.

## Data Availability

The data that support the findings of this study are available on request from the corresponding author. The data are not publicly available due to privacy or ethical restrictions.
