# Supplementary material for: Real‐Time Feedback Strategically Regulates Optoelectronics for Customized Optogenetic Spinal Cord Regeneration
Source: Exploration (Beijing). 2026 Apr 9;6(2):20250138. doi: 10.1002/EXP.20250138 (PMC13094529; doi:10.1002/EXP.20250138)
Supplement: Supplementary file 1 — Supporting File 1: exp270159‐sup‐0001‐SuppMat.docx. [file EXP2-6-20250138-s001.docx]

**Inventory of Supplemental Information**

**I Supplemental Figures and Legends**

Figure S1, related to Figure 1.

Figure S2, related to Figure 1.

Figure S3, related to Figure 1.

Figure S4, related to Figure 2.

Figure S5, related to Figure 2.

Figure S6, related to Figure 3.

Figure S7, related to Figure 3.

Figure S8.

Figure S9, related to Figure 4.

Figure S10, related to Figure 5.

Figure S11, related to Figure 6 and Figure 7.

**II Supplemental Tables**

Table S1. Summary of a series of implantable optogenetic bioelectronics. Related to Introduction.

Table S2. Thermal parameters of materials. Related to Figure 2.

Table S3. Mechanical properties of materials. Related to Figure 3.

Table S4. Antibodies used in WB and IF. Related to Figures 6, 7 and S6.

**I Supplemental Figures and Legends**


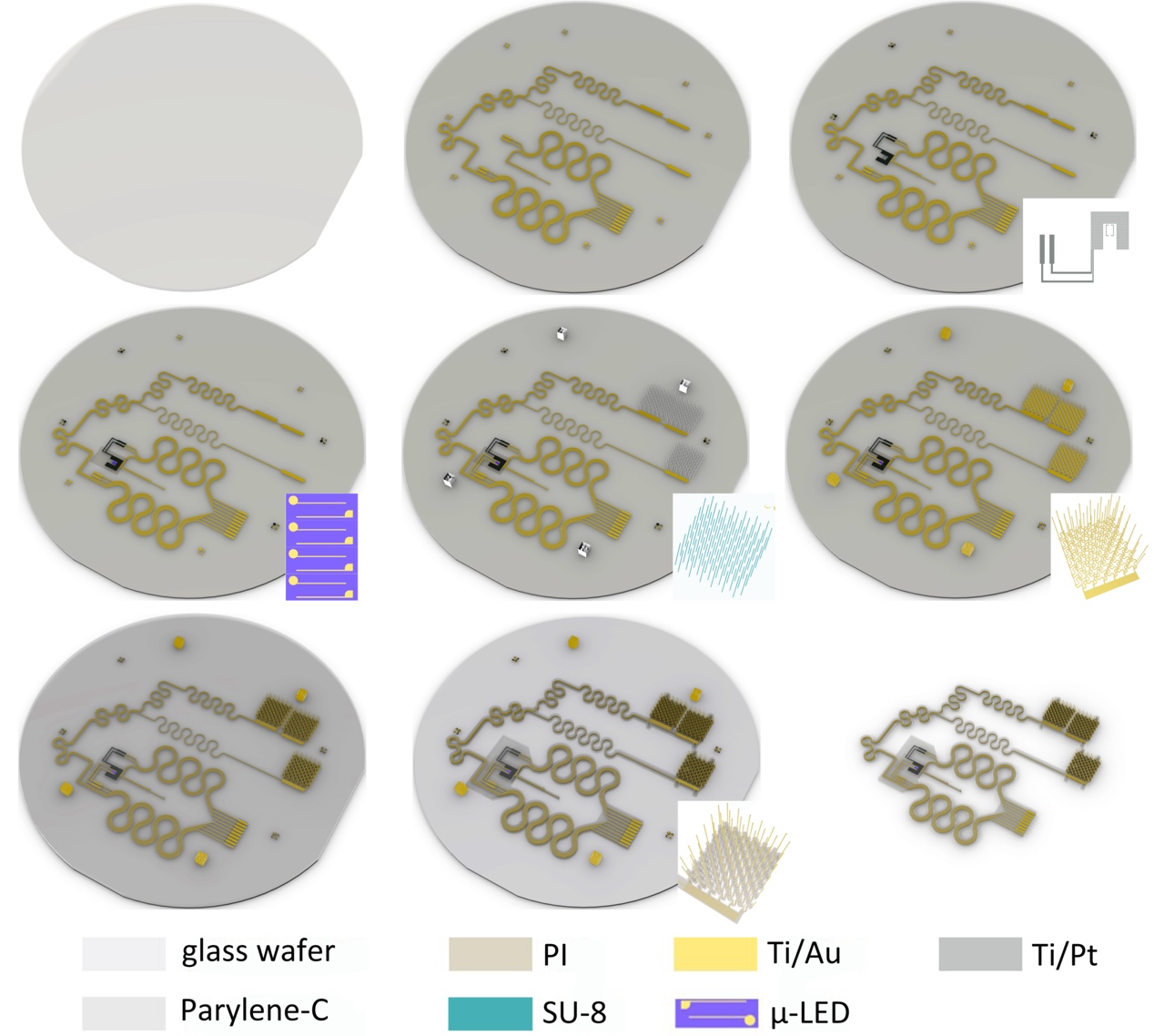


**Figure S1**, related to Figure 1. Representative soft, stretchable, fully implantable, programmable, triple-threaded miniaturized optoelectronic system manufacturing and preparation process.

In brief, polymethyl methacrylate (PMMA) and polyimide (PI) substrate (10μm) were prepared on glass wafer. Sputtering titanium and gold (30 nm / 200 nm) layer was used to graphically prepare the underlying circuit by photolithographic etching. A resistive temperature sensor was obtained by sputtering titanium platinum layer (30nm/150nm) with ultrasonic assisted stripping. A PDMS film (50μm) with a square hole was cut by laser and attached to the PI surface. A small amount of PI was poured into the hole and rotated evenly. The Polydimethylsiloxane (PDMS) were removed and heated at 90℃ for 10s on the heating plate, with the μ-LED transferred onto the exact position. The SU-8 microneedle array was prepared by a specific process; Sputtering copper (200nm) spin coated photoresist (AZ5214E) and full exposure, spin coated the above photoresist again, patterned exposure, appropriately extended development time to form a depression zone, copper corrosion further into the depression zone to form a trapezoidal section structure more suitable for lift-off three-dimensional structure. Sputtering titanium and gold (30 nm/200 nm) layer for stripping; Then remove the remaining copper and photoresist. Encapsulation by Parylene coating; Sputtered copper (200nm) photolithographic etching, exposed the pad, microneedles, and the pores between them. RIE was carried out with copper as a mask until the surface was removed along with the Parylene. Finally, the outline of the device is obtained by laser cutting, and the final device is obtained by using acetone to penetrate the PI substrate and remove PMMA.


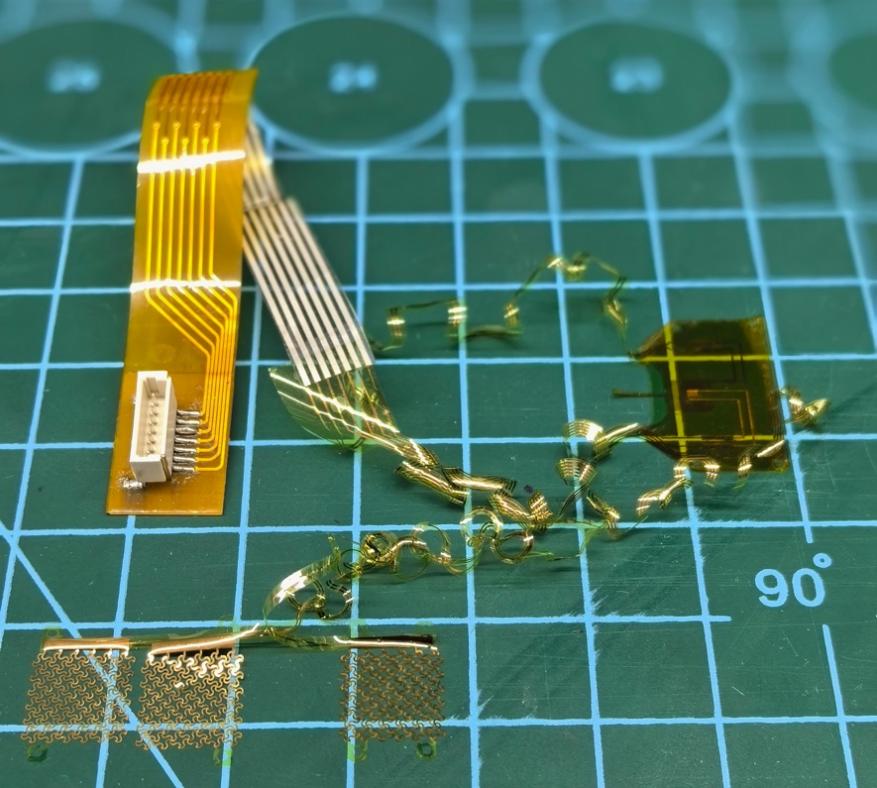


**Figure S2**, related to Figure 1. Actual image of the soft, multimodal sensing and programmable miniaturized optogenetic bioelectronic system.


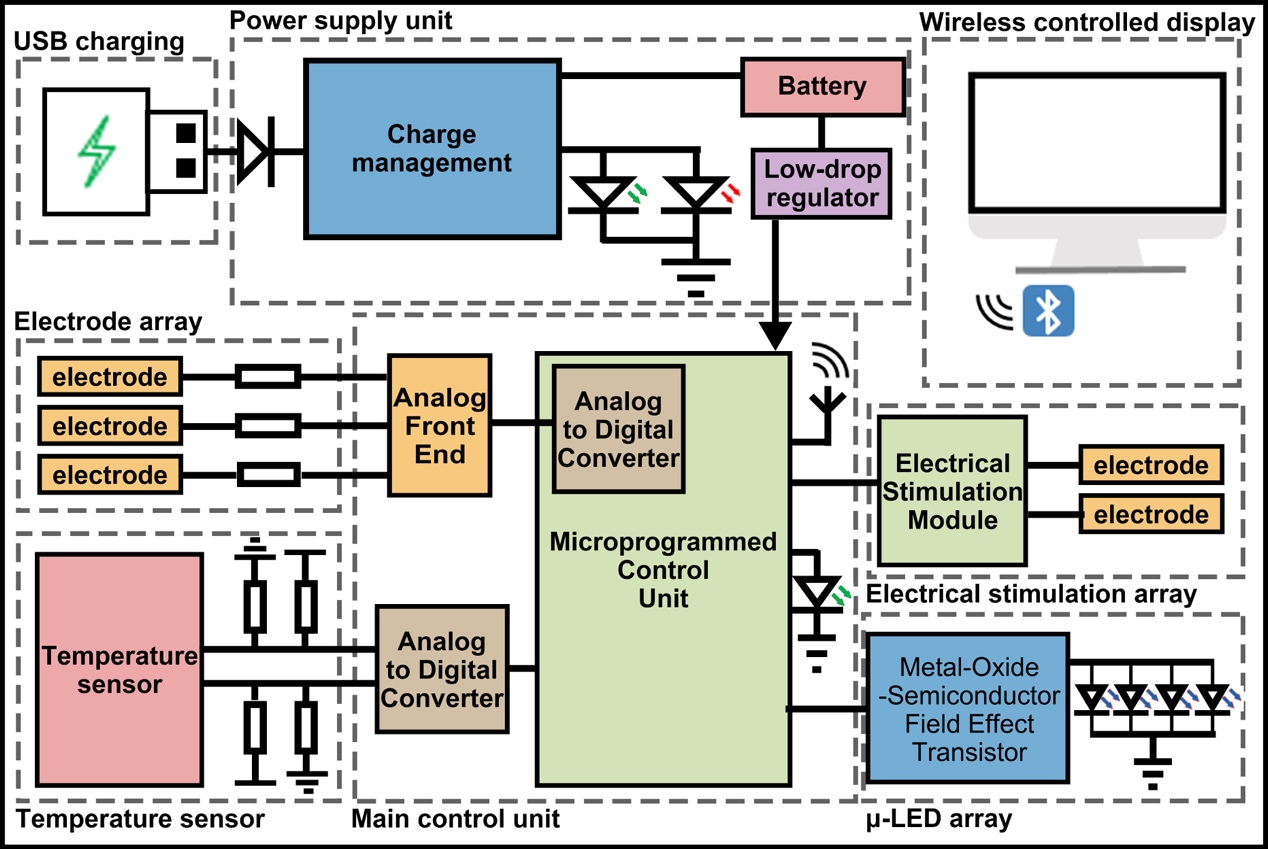


**Figure S3**, related to Figure 1. Electrical circuit diagram of the overall custom-designed external control board.


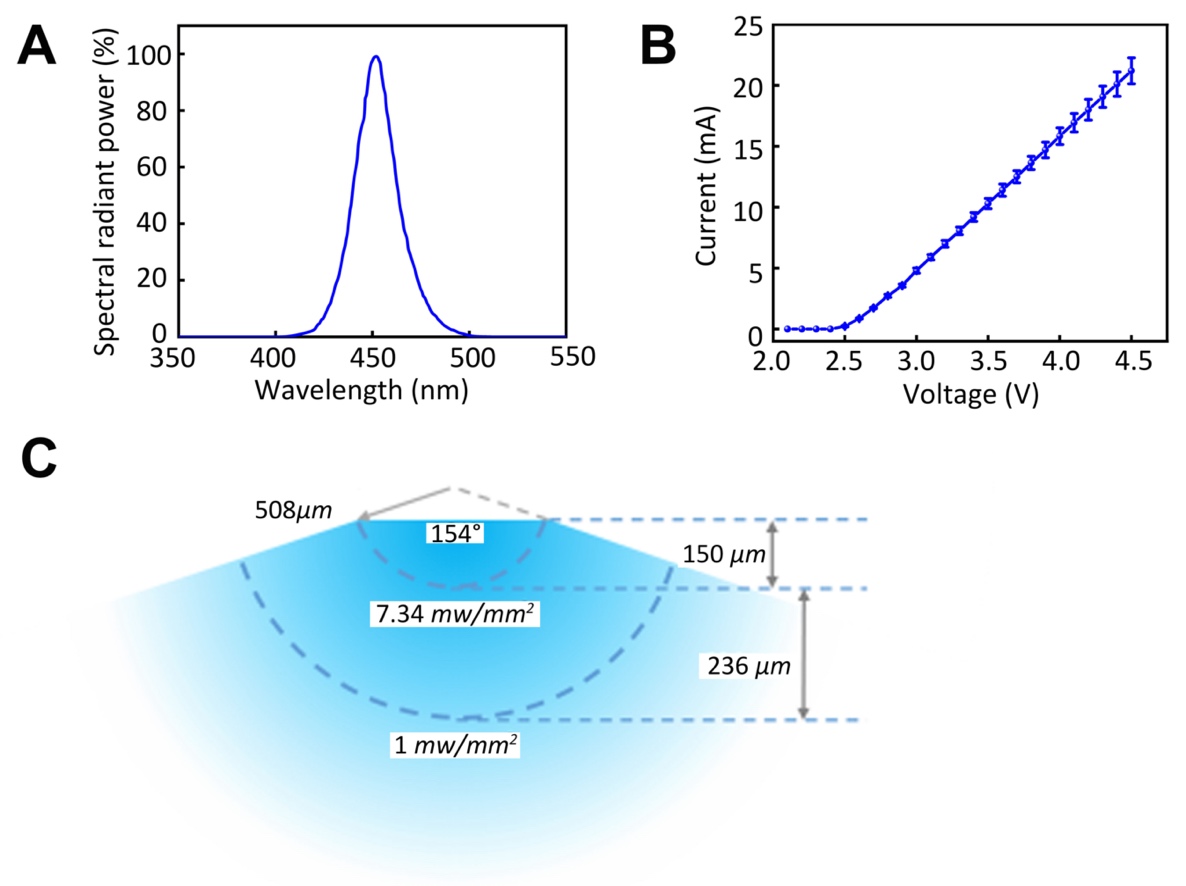


**Figure S4**, related to Figure 2. Optical characteristics of the optoelectronic system.

(A) Wavelength result of the μ-LED array.

(B) Current-voltage characteristic of the μ-LED array.

(C) Typical 75% PWM illumination simulation results, the optical power density is 7.34 mW/mm^2^ on the surface of the spinal cord, and the power density decreases to 1 mW/mm^2^ at 236 μm depth.


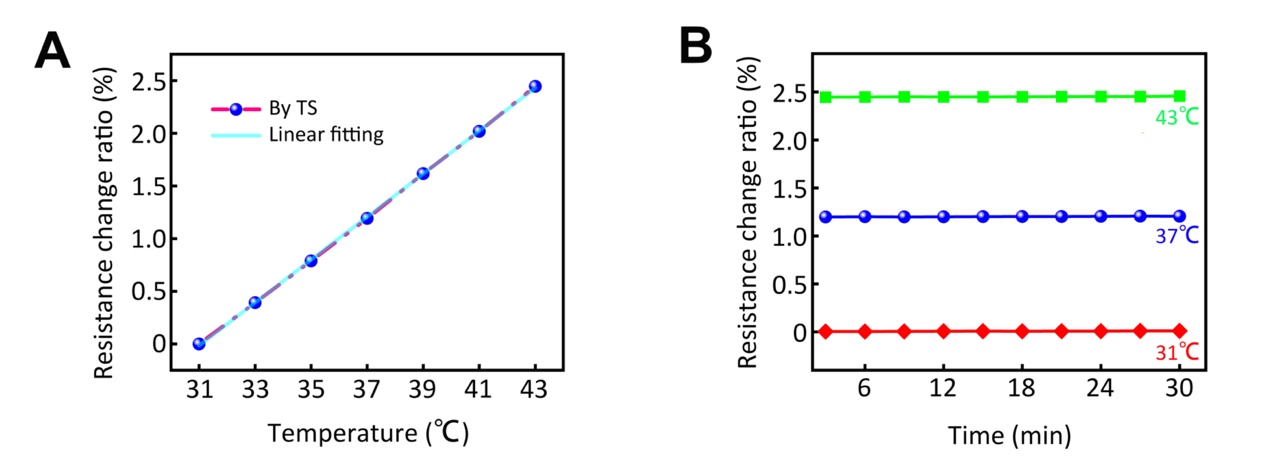


**Figure S5**, related to Figure 2. Thermal characteristics of the optoelectronic system.

(A) Standardized resistance changes ratio of the temperature sensor increased linearly with temperature changes.

(B) Stabilized temperature sensor shows almost no changes of the resistance change ratio at 31°C, 37°C and 43°C.


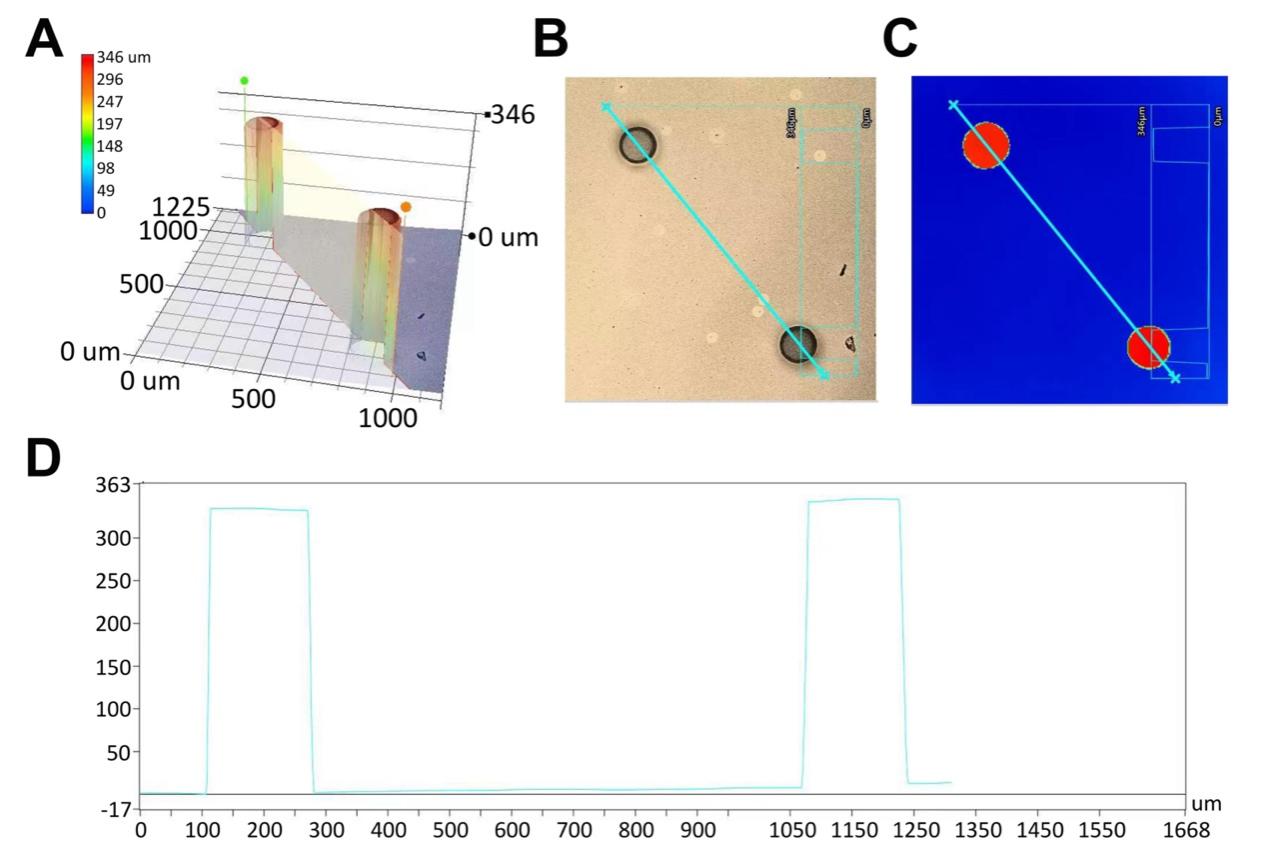


**Figure S6**, related to Figure 3. Electronic characteristics of the optoelectronic system.

(A) Morphology of the electrode array with 3D reconstruction by optical microscope (VHX-6000, Keyence)

(B) Plan view of the electrode array.

(C) Infrared plan view of the electrode array

(D) Quantitative results of the electrode array.


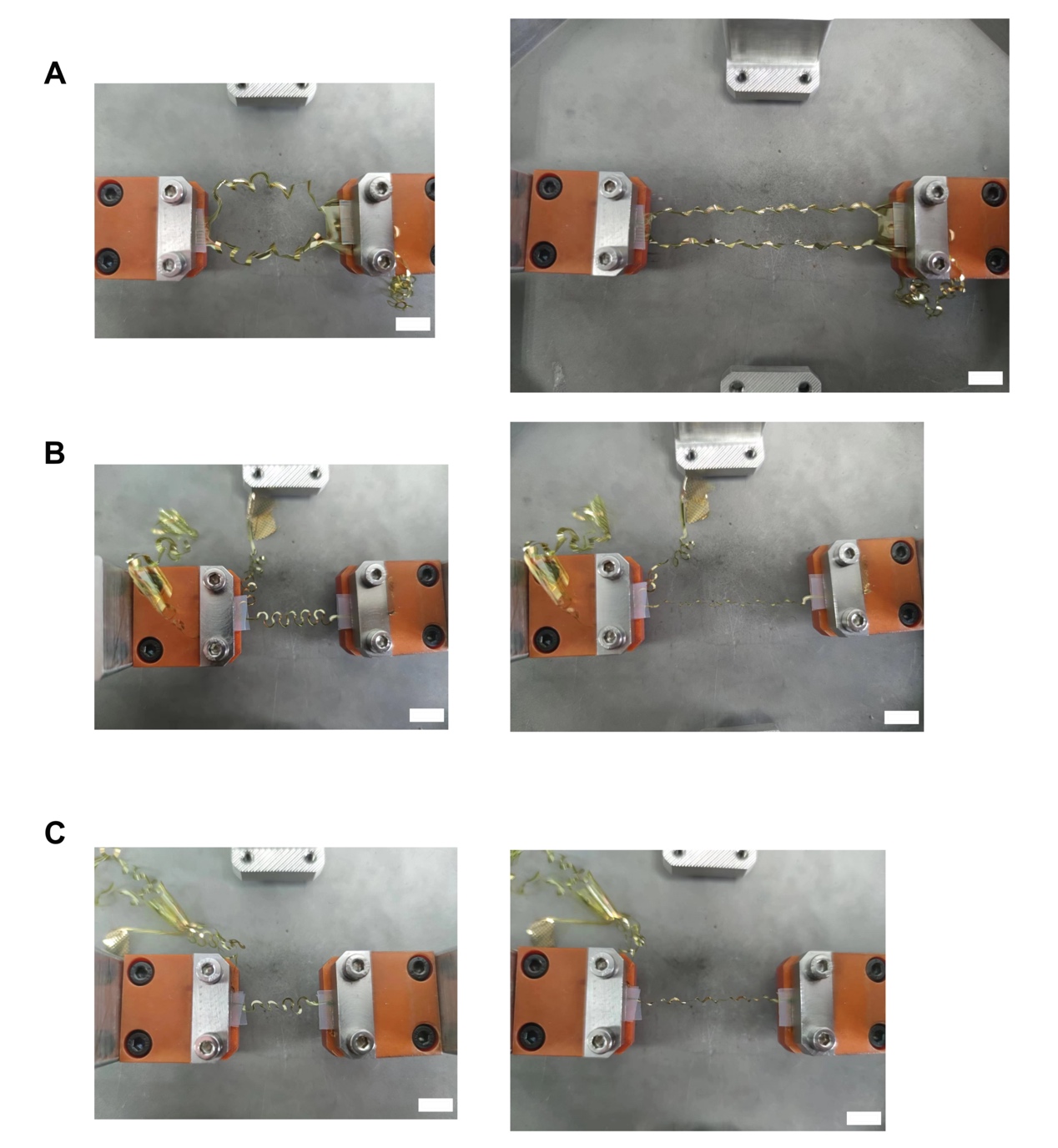


**Figure S7**, related to Figure 3. Stretch characteristics of the optoelectronic system.

(A) Actual stretch results of the serpentine circuits, which is corresponding to a in Figure 3E. Scale bar = 1000 μm.

(B) Actual stretch results of the serpentine circuits, which is corresponding to b in Figure 3E. Scale bar = 1000 μm.

(C) Actual stretch results of the serpentine circuits, which is corresponding to c in Figure 3E. Scale bar = 1000 μm.


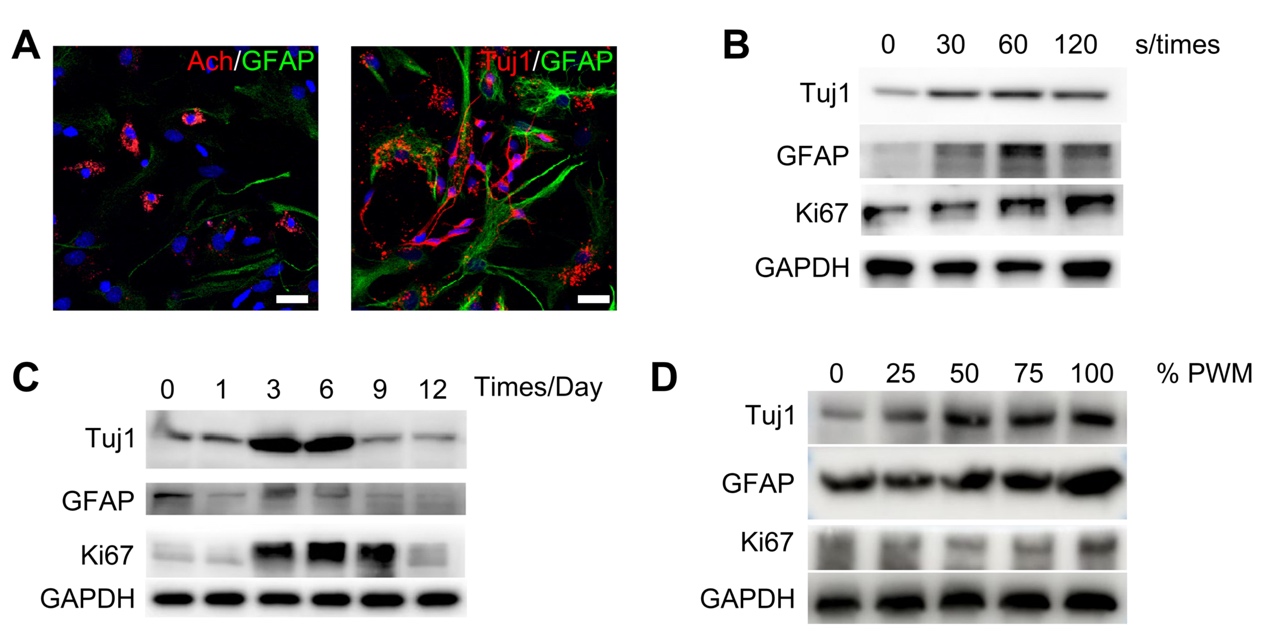


**Figure S8**, *in vitro* results of the hChR2-rSNSCs with neuron differentiation and proliferation capacity to evaluate the frequency, intensity, and duration of the device illumination.

(A) hChR2-rSNSCs show promising neuron differentiation capacity and is capable of differentiation into acetylcholine neurons. Scale bar= 100 μm.

(B) The relative protein expression of hChR2-rSNSCs differentiation and proliferation under the illumination of 470 nm μ-LED array for 0, 30, 60 120 seconds every time.

(C) The relative protein expression of hChR2-rSNSCs differentiation and proliferation under the illumination of 470 nm μ-LED array for 0, 1, 3, 6, 9, 12 times every day.

(D) The relative protein expression of hChR2-rSNSCs differentiation and proliferation under the illumination of 470 nm μ-LED array for 0%, 25%, 50%, 75% and 100% PWM every time.


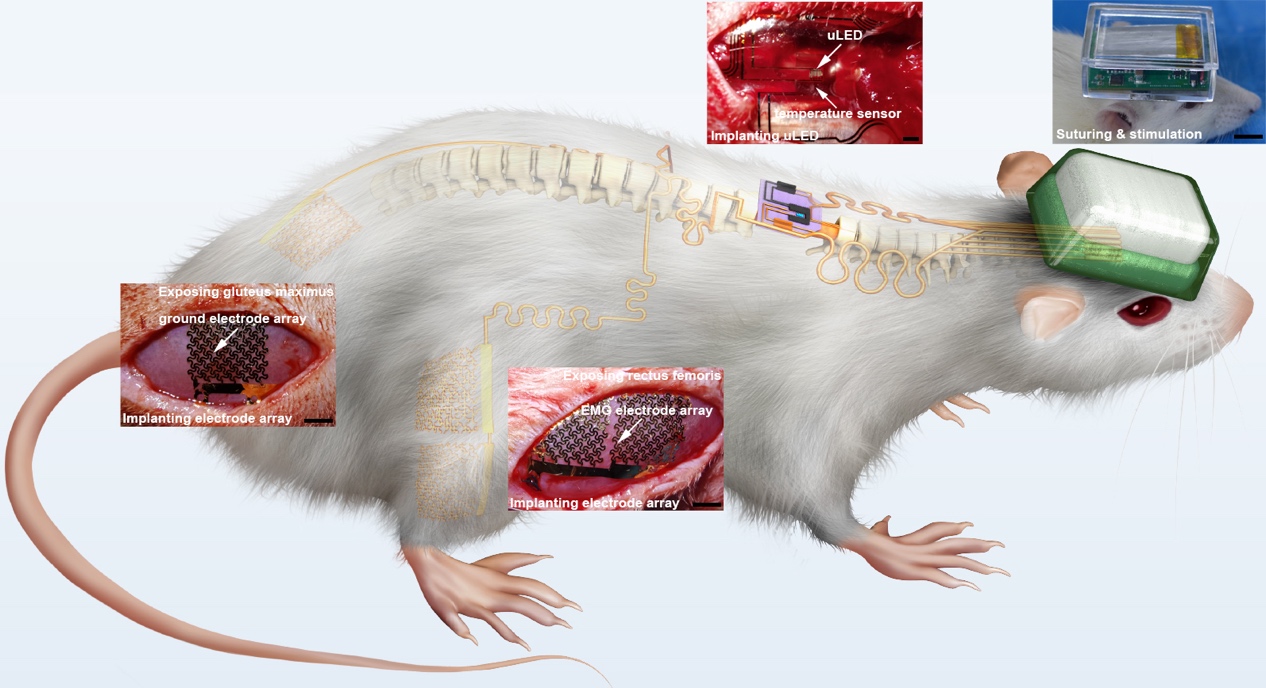


**Figure S9**, related to Figure 4. The total components within an intact rat model at different locations.


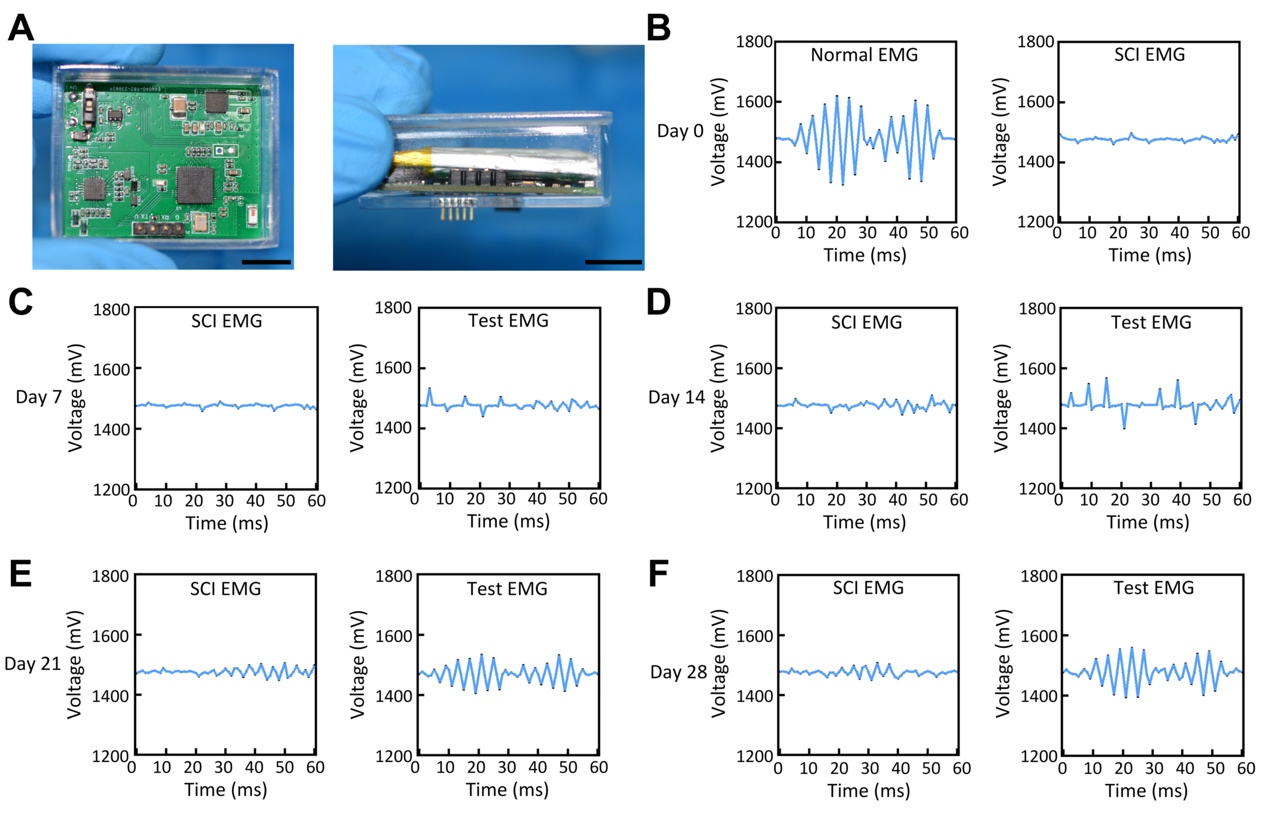


**Figure S10**, related to Figure 5. EMG results to standardize the recovery condition of the test rats with identical intensity of electrical stimulation.

(A) Typical top and side views of the ECB.

(B) Typical EMG results of the normal rat and the SCI rat at Day 0.

(C) Typical EMG results of the SCI rat and the most well performed rat at Day 7.

(D) Typical EMG results of the SCI rat and the most well performed rat at Day 14.

(E) Typical EMG results of the SCI rat and the most well performed rat at Day 21.

(F) Typical EMG results of the SCI rat and the most well performed rat at Day 28.


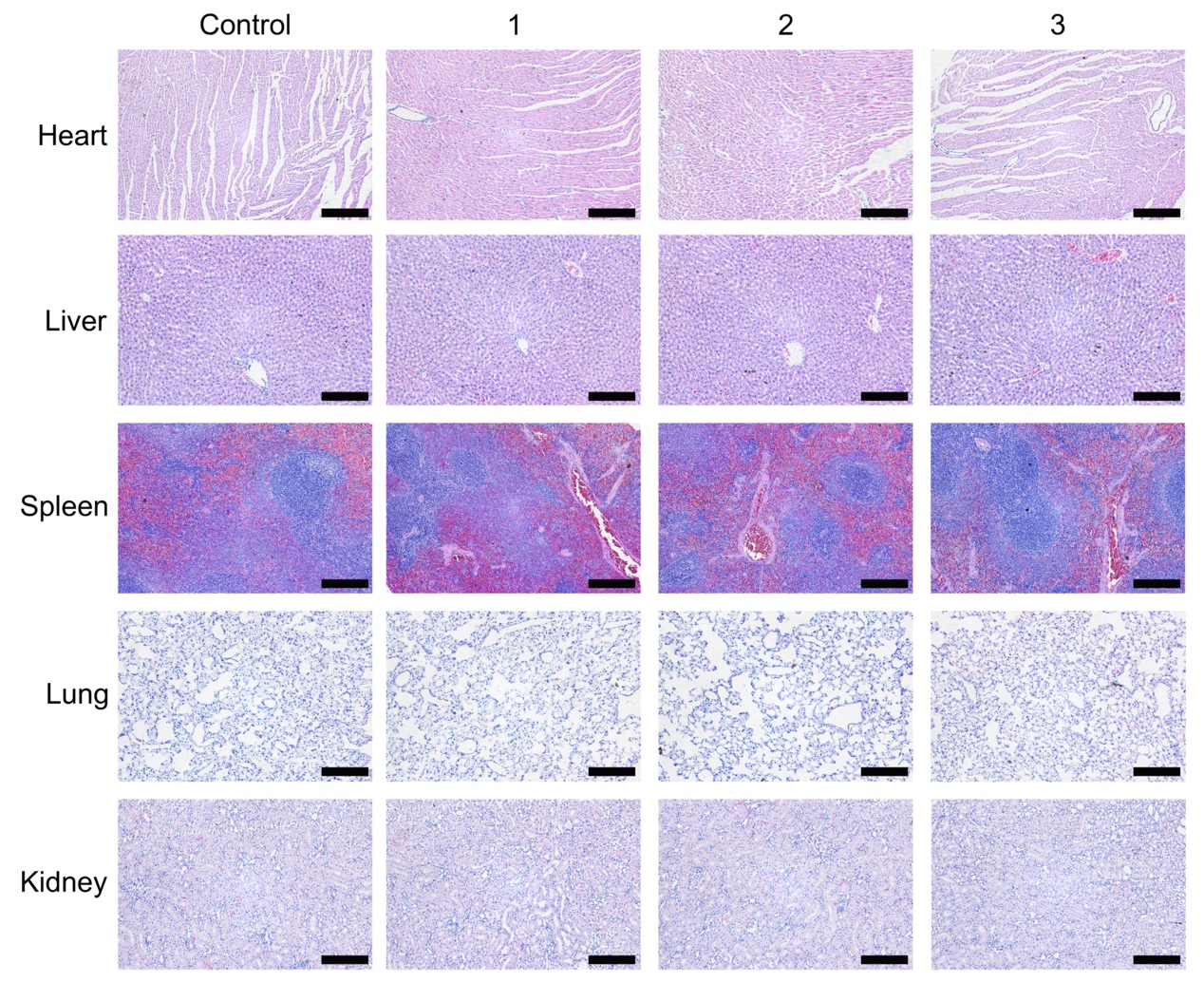


**Figure S11**, related to Figure 6 and Figure 7. Toxicity test of optoelectronic system implantation with the critical organs in rats. Scale bar = 1000 μm.

**II Supplemental Tables**

**Table S1.**


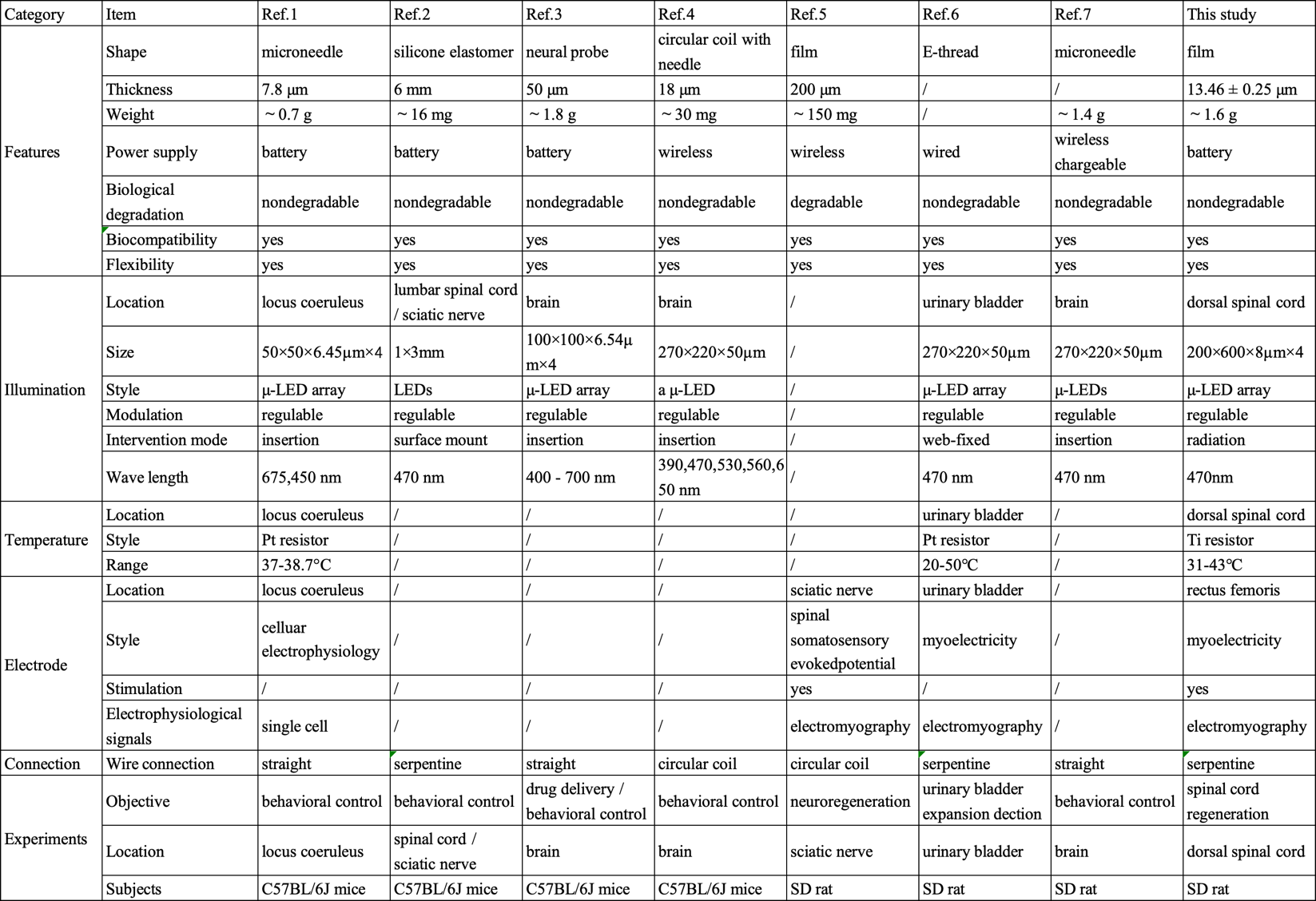


Reference：

1. Kim, T. I.; McCall, J. G.; Jung, Y. H.; Huang, X.; Siuda, E. R.; Li, Y.; Song, J.; Song, Y. M.; Pao, H. A.; Kim, R. H.; Lu, C.; Lee, S. D.; Song, I. S.; Shin, G.; Al-Hasani, R.; Kim, S.; Tan, M. P.; Huang, Y.; Omenetto, F. G.; Rogers, J. A.; Bruchas, M. R., Injectable, Cellular-scale Optoelectronics with Applications for Wireless Optogenetics. *Science* **2013,** *340* (6129), 211-6.

2. Park, S. I.; Brenner, D. S.; Shin, G.; Morgan, C. D.; Copits, B. A.; Chung, H. U.; Pullen, M. Y.; Noh, K. N.; Davidson, S.; Oh, S. J.; Yoon, J.; Jang, K. I.; Samineni, V. K.; Norman, M.; Grajales-Reyes, J. G.; Vogt, S. K.; Sundaram, S. S.; Wilson, K. M.; Ha, J. S.; Xu, R.; Pan, T.; Kim, T. I.; Huang, Y.; Montana, M. C.; Golden, J. P.; Bruchas, M. R.; Gereau, R. W. t.; Rogers, J. A., Soft, Stretchable, Fully Implantable Miniaturized Optoelectronic Systems for Wireless Optogenetics. *Nat Biotechnol* **2015,** *33* (12), 1280-1286

3. Jeong, J. W.; McCall, J. G.; Shin, G.; Zhang, Y.; Al-Hasani, R.; Kim, M.; Li, S.; Sim, J. Y.; Jang, K. I.; Shi, Y.; Hong, D. Y.; Liu, Y.; Schmitz, G. P.; Xia, L.; He, Z.; Gamble, P.; Ray, W. Z.; Huang, Y.; Bruchas, M. R.; Rogers, J. A., Wireless Optofluidic Systems for Programmable In Vivo Pharmacology and Optogenetics. *Cell* **2015,** *162* (3), 662-74.

4. Shin, G.; Gomez, A. M.; Al-Hasani, R.; Jeong, Y. R.; Kim, J.; Xie, Z.; Banks, A.; Lee, S. M.; Han, S. Y.; Yoo, C. J.; Lee, J. L.; Lee, S. H.; Kurniawan, J.; Tureb, J.; Guo, Z.; Yoon, J.; Park, S. I.; Bang, S. Y.; Nam, Y.; Walicki, M. C.; Samineni, V. K.; Mickle, A. D.; Lee, K.; Heo, S. Y.; McCall, J. G.; Pan, T.; Wang, L.; Feng, X.; Kim, T. I.; Kim, J. K.; Li, Y.; Huang, Y.; Gereau, R. W. t.; Ha, J. S.; Bruchas, M. R.; Rogers, J. A., Flexible Near-Field Wireless Optoelectronics as Subdermal Implants for Broad Applications in Optogenetics. *Neuron* **2017,** *93* (3), 509-521.e3.

5. Koo, J.; MacEwan, M. R.; Kang, S. K.; Won, S. M.; Stephen, M.; Gamble, P.; Xie, Z.; Yan, Y.; Chen, Y. Y.; Shin, J.; Birenbaum, N.; Chung, S.; Kim, S. B.; Khalifeh, J.; Harburg, D. V.; Bean, K.; Paskett, M.; Kim, J.; Zohny, Z. S.; Lee, S. M.; Zhang, R.; Luo, K.; Ji, B.; Banks, A.; Lee, H. M.; Huang, Y.; Ray, W. Z.; Rogers, J. A., Wireless Bioresorbable Electronic System Enables Sustained Nonpharmacological Neuroregenerative Therapy. *Nat Med* **2018,** *24* (12), 1830-1836.

6. Jang, T. M.; Lee, J. H.; Zhou, H.; Joo, J.; Lim, B. H.; Cheng, H.; Kim, S. H.; Kang, I. S.; Lee, K. S.; Park, E.; Hwang, S. W., Expandable and Implantable Bioelectronic Complex for Analyzing and Regulating Real-time Activity of the Urinary Bladder. *Sci Adv* **2020,** *6* (46).

7. Kim, C. Y.; Ku, M. J.; Qazi, R.; Nam, H. J.; Park, J. W.; Nam, K. S.; Oh, S.; Kang, I.; Jang, J. H.; Kim, W. Y.; Kim, J. H.; Jeong, J. W., Soft Subdermal Implant Capable of Wireless Battery Charging and Programmable Controls for Applications in Optogenetics. *Nat Commun* **2021,** *12* (1), 535.

**Table S2.** Thermal parameters of materials. Related to Figure 2

|  | Density *ρ (toone/mm3)* | Specific heat *c (mJ/tonne・K)* | Thermal conductivity *k (mW/mm・K)* |
| --- | --- | --- | --- |
| Substrate and encapsulation layer | 1.34×10^-9 | 1.04×10^9 | 0.22 |
| μ-LED | 2.3016×10^-9 | 7.13×10^8 | 124 |
| Vertebral plate | 1.908×10^-9 | 1.313×10^9 | 0.32 |
| Spinal cord | 1.075×10^-9 | 3.63×10^9 | 0.51 |
| Mean rat parameter | 1.015×10^-9 | 3.47×10^9 | 0.5 |

**Table S3.** Mechanical properties of materials. Related to Figure 3

|  | Density (g/cm3) | Young modulus (MPa) | Poisson ratio (MPa) | Yield strength (MPa) | Yield elongation |
| --- | --- | --- | --- | --- | --- |
| PI | 1.42 | 300 | 0.34 | 60-80 | 5% |
| Au | 19.32 | 79500 | 205 | 220 | 4-6% |
| Parylene | 1.289 | 3200 | 55.2 | 68.9 | 2.90% |

**Table S4**. Antibodies used in WB and IF. Related to Figures 6, 7 and S6

| Product name | Catalog | Company |
| --- | --- | --- |
| Anti-Neurofilament heavy polypeptide antibody | ab207176 | abcam |
| Anti-MAP2 antibody | ab11267 | abcam |
| Anti-Choline Acetyltransferase antibody | ab181023 | abcam |
| Anti-CD68 antibody | ab283654 | abcam |
| Anti-GFAP antibody | ab68428 | abcam |
| Anti-beta III Tubulin antibody | ab18207 | abcam |
| Anti-Ki67 antibody | ab16667 | abcam |
| Anti-GAPDH antibody | ab181602 | abcam |
